# A protein subunit vaccine elicits a balanced immune response that protects against *Pseudomonas* pulmonary infection

**DOI:** 10.1038/s41541-023-00618-w

**Published:** 2023-03-14

**Authors:** Debaki R. Howlader, Sayan Das, Ti Lu, Rahul Shubhra Mandal, Gang Hu, David J. Varisco, Zackary K. Dietz, Siva Sai Kumar Ratnakaram, Robert K. Ernst, William D. Picking, Wendy L. Picking

**Affiliations:** 1 Department of Pharmaceutical Chemistry, University of Kansas, Lawrence, KS 66047 USA; 2 Department of Microbial Pathogenesis, University of Maryland, Baltimore, MD 21201 USA; 3 Perelman School of Medicine, University of Pennsylvania, Philadelphia, PA 19104 USA; 4 Present Address: Department of Veterinary Pathobiology, University of Missouri, Columbia, MO 65211 USA

**Keywords:** Protein vaccines, Bacterial infection

## Abstract

The opportunistic pathogen *Pseudomonas aeruginosa* (Pa) causes severe nosocomial infections, especially in immunocompromised individuals and the elderly. Increasing drug resistance, the absence of a licensed vaccine and increased hospitalizations due to SARS-CoV-2 have made Pa a major healthcare risk. To address this, we formulated a candidate subunit vaccine against Pa (L-PaF), by fusing the type III secretion system tip and translocator proteins with LTA1 in an oil-in-water emulsion (ME). This was mixed with the TLR4 agonist (BECC438b). Lung mRNA sequencing showed that the formulation activates genes from multiple immunological pathways eliciting a protective Th1-Th17 response following IN immunization. Following infection, however, the immunized mice showed an adaptive response while the PBS-vaccinated mice experienced rapid onset of an inflammatory response. The latter displayed a hypoxic lung environment with high bacterial burden. Finally, the importance of IL-17 and immunoglobulins were demonstrated using knockout mice. These findings suggest a need for a balanced humoral and cellular response to prevent the onset of Pa infection and that our formulation could elicit such a response.

## Introduction

*Pseudomonas aeruginosa* (Pa)^1,2^ is a significant cause of nosocomial infections in health care settings. Immunocompromised individuals, burn victims, cystic fibrosis patients, and cancer patients are at a further elevated risk of infection by Pa^3,4^. A wide range of virulence factors are used by this opportunistic pathogen to initiate infection and subsequently adapt to its environment leading to chronic biofilm formation and acquisition of drug-resistance^5^. Recent reports have also acknowledged a significant increase in the identification of Multi-Drug Resistant (MDR) Pa strains and Extremely Drug Resistant (XDR) Pa strains^5^. The Centers for Disease Control and Prevention estimated attributable healthcare costs of $757 M with nearly 32,600 cases of MDR Pa infection in 2017^6^. Furthermore, ventilator-associated pneumonia (VAP) caused by Pa is a common occurrence in 3–5% of adults ventilated for more than 2 days^7^. Likewise, Pa infection was the most common infection in military troops in 2016^2^. Although certain diseases and occupational health hazards increase the risking for Pa infection, aging is the most common factor responsible for a lethal Pa infection^2^.

Faced with the elevated risks posed by MDR and XDR Pa, the most efficient way to move forward is to develop a broadly protective prophylactic vaccine to prevent the onset of infection. Several approaches have been used in the past, but no licensed Pa vaccine has reached the market. Vaccine formulation incorporating LPS O-antigen^8^, outer membrane proteins^9^, live-attenuated vaccines^10,11^, or whole cell vaccines^12^ are being developed, yet there is still no licensed Pa vaccine. Toward this end, type-III secretion system (T3SS) proteins present at the exposed tip of the T3SS apparatus (T3SA) needle have been tested for their efficacy recently with promising results^13–16^.

Our group previously demonstrated that a fusion of LTA-1 (the active moiety of the A subunit from the heat-labile enterotoxin of Enterotoxigenic *Escherichia coli*) with the Pa T3SA needle tip protein (PcrV) and first translocator protein (PopB) to give L-PaF was an effective vaccine against Pa infection in BALB/c mice^17^. In this study, L-PaF was used at multiple doses, with and without additional adjuvants to determine its effectiveness in protecting against Pa infection in the more genetically and immunologically diverse CD-1 mouse model. The formulations’ effects on cellular and humoral immune responses were then evaluated in terms of immune correlates. Lung mRNA seq analysis was also done to determine the immune pathways activated by the vaccine and how they compare to those activated by the actual Pa infection in non-immunized mice. Lastly, the effects of IL-17 and immunoglobulins, thought to be important for rapid Pa clearance prior to establishing chronic infection, were evaluated in vivo using *il17*^−*/−*^ and muMt^-^ knockout (KO) mice.

## Results

### Specific vaccine formulations induce serum immunoglobulins with high serum opsonophagocytic activity

**Fig. 1:**
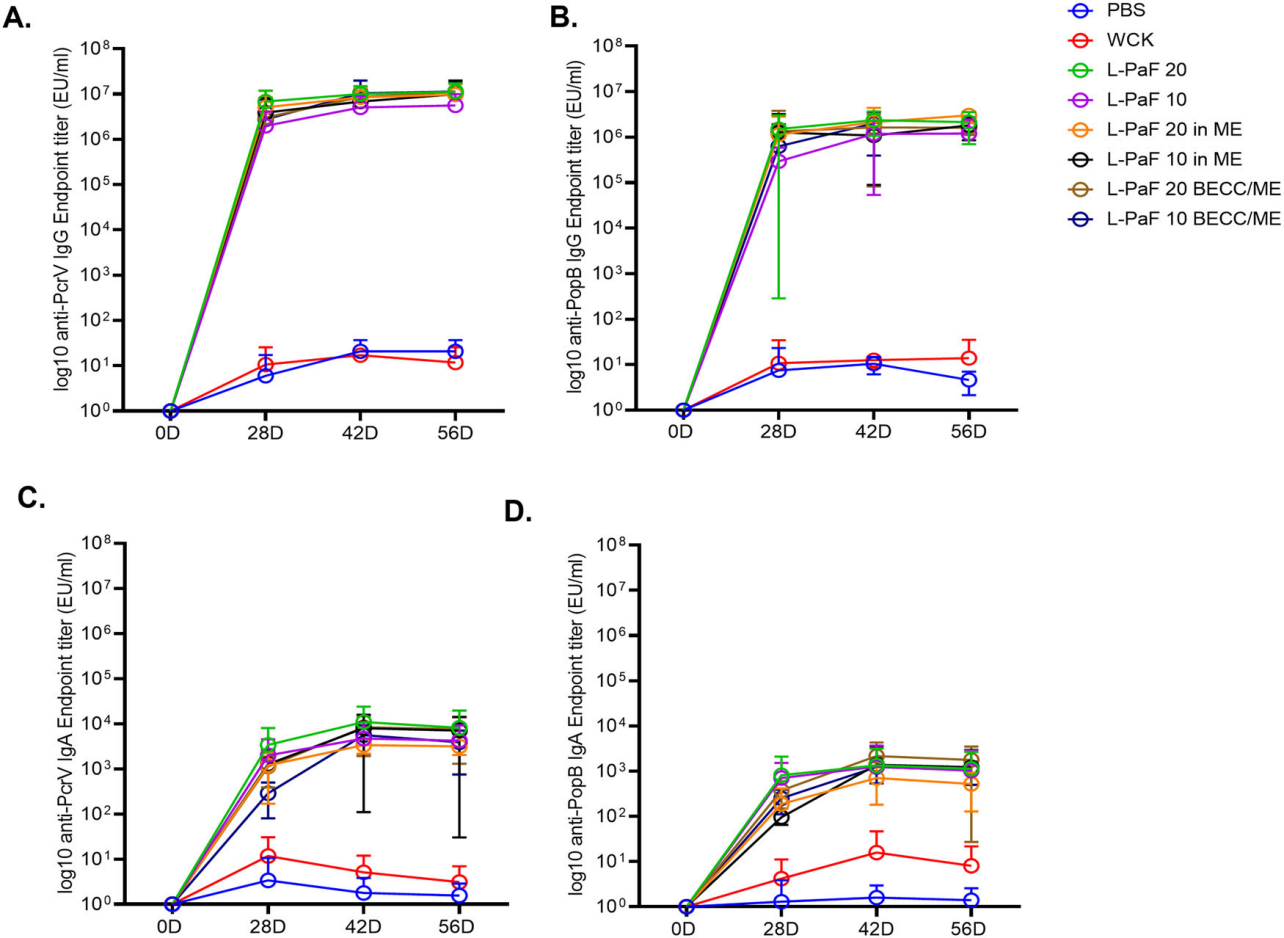
Kinetics of serum IgG and IgA. CD-1 mice were vaccinated on days 0, 14 and 28 and their sera were assessed for anti-PcrV and anti-PopB immunoglobulins as a function of time and boosting. Anti-PcrV IgG (**A**) and IgA (**C**), along with anti-PopB IgG (**B**) and IgA (**D**), are shown. Individual titers are represented as EU/ml. Each point denotes a mean and error bars represent SD for each group (*n* = 10 mice/group).

Based on our previous work with L-PaF^13^, CD-1 mice were vaccinated with two concentrations of L-PaF (20 or 10 µg) alone, with ME or with BECC/ME. PBS was used as a vaccinated negative control. Mice were immunized intranasally (IN) starting at 6–8 weeks of age on days 0, 14 and 28. Regardless of formulation, L-PaF elicited significant systemic humoral immune responses (i.e., IgG and IgA) against both PcrV and PopB while PBS and Whole Cell Killed Pa (WCK) did not (Fig. 1). IgG subclasses were also measured and all L-PaF formulations were found to be significantly higher for all subclasses than the PBS control. IgG1 was the major subtype observed, followed by IgG2a and IgG3 (Supplementary Fig. 1). Significant differences in the generation of IgG1, IgG2a, and IgG3 were observed for the 10 μg L-PaF BECC/ME mice relative to the PBS group and 20 μg L-PaF BECC/ME produced significantly higher IgG2a and IgG3 relative to PBS.

**Fig. 2:**
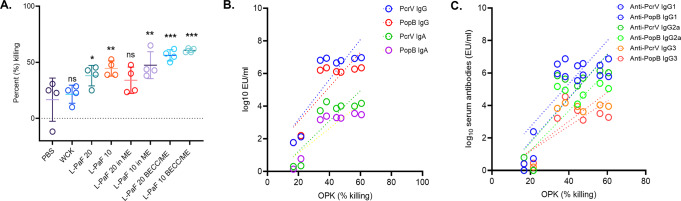
Serum immunoglobulins possess opsonophagocytic killing (OPK) activity. The day 42 sera from Fig. 1 were heat-treated at 56 °C for 30 min for subsequent use in OPK assays. **A** Relative OPK activities are shown for sera collected from each CD-1 mouse group are shown. **B** A correlation between OPK and either anti-PcrV or anti-PopB immunoglobulin titers is shown. **C** A correlation between OPK and specific subclasses of IgG against PcrV and PopB is shown. The points in (**A**) represent individual OPK values obtained from the experiment (error bars represent SD for each group), whereas the points in (**B**) and (**C**) represent correlation values obtained between OPK and pooled D42 serum. Pearson’s r coefficient and simple linear regression (95% confidence level) were calculated for (**B**) and (**C**). *p < 0.05, **p < 0.01, ***p < 0.001. Exact *p* values can be found in Supplementary Tables 1, and 2.

Opsonophagocytic killing (OPK) assays were performed as an in vitro functional assay to determine whether the secreted immunoglobulins promote specific macrophage killing of Pa (Fig. 2). In comparison to the PBS vaccinated mice, all other groups possessed significantly higher OPK activity except for the WCK and 20 μg L-PaF/ME groups. Both doses of L-PaF alone and with ME possessed 34–47% OPK activity (Fig. 2A) with the L-PaF formulations containing BECC438b and ME showing the highest killing ability with 10 µg and 20 μg L-PaF BECC/ME exhibiting 61 and 55% OPK activity, respectively.

To determine whether the observed OPK levels correlate with immunoglobulin titers for day 42 serum, a correlation analysis was carried out. OPK values positively correlated with the amount of serum immunoglobulins present (*r* > 0.80 to <0.87, *p* < 0.01) (Fig. 2B and Supplementary Table 1). This was also positively correlated with the amounts of IgG sub-types (Fig. 2C and Supplementary Table 2). OPK activity appears to be an important determinant for reducing infection in vivo since mucoid Pa strains have been reported to be more resistant to non-opsonic phagocytosis than their non-mucoid counterparts^1^. Immunoglobulins present in the serum participate in OPK activity to kill mucoid Pa (mPa08-31).

### PcrV and PopB can stimulate Th1 and Th17 cells in the lung: a potentially important determinant of protection

**Fig. 3:**
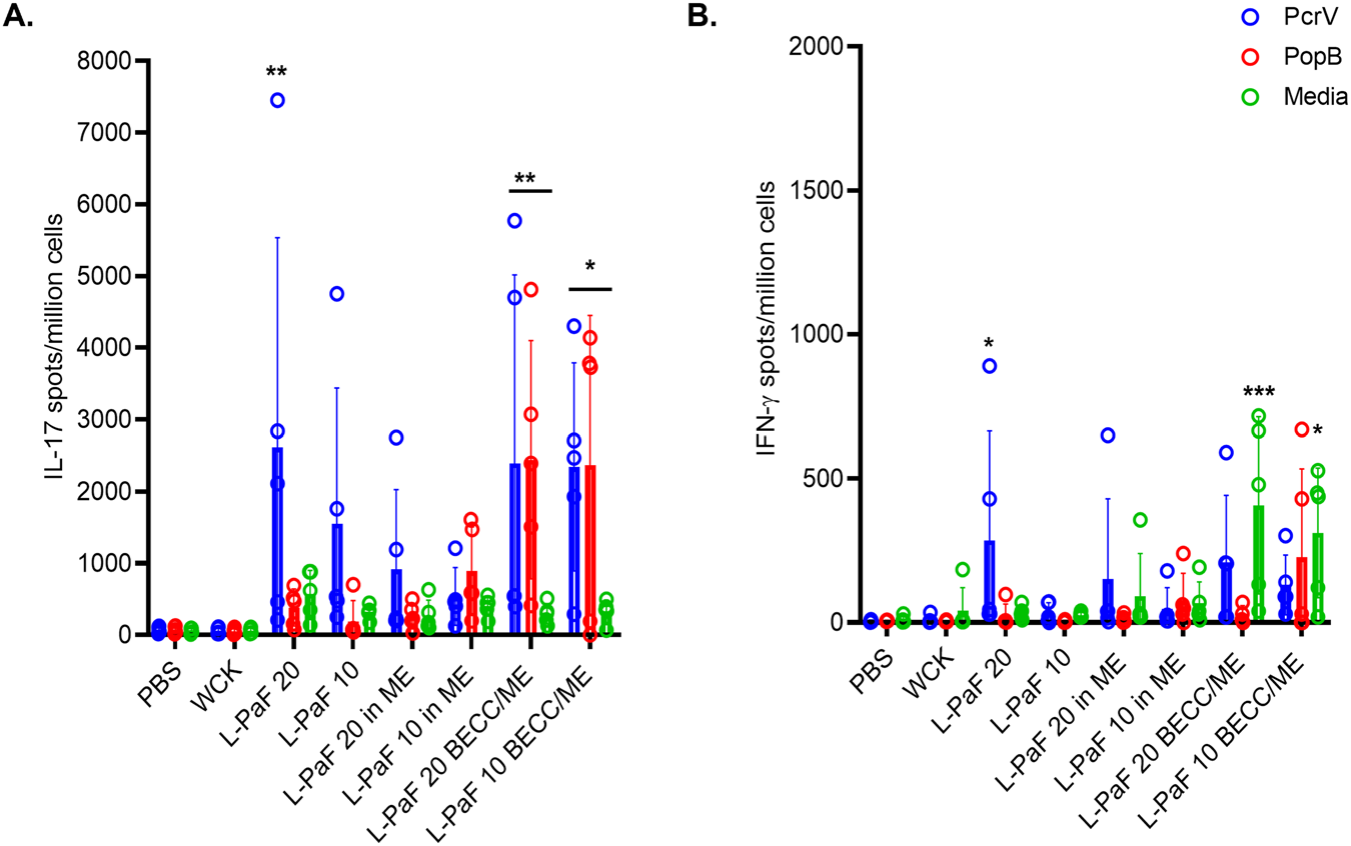
IL-17 and IFN-γ secretion following ex vivo stimulation of lung cells. Cell suspensions were prepared and treated with either PcrV or PopB or left untreated (media). IL-17 (**A**) and IFN-γ (**B**) secreting cells were quantified after 24 h stimulation and are shown as secreting cells per million total cells. Values were plotted as individual points ± SD (*n* = 5/group). Error bars represent SD. Statistical significance was calculated by comparing PBS vaccinated group with each of the other vaccinated groups using Dunnett’s test. *p < 0.05, **p < 0.01, ***p < 0.001.

At 56 days post the first immunization (DPIm), lung cells were isolated and stimulated with either PcrV or PopB and the cytokine secreting cells were enumerated using ELISpot (ImmunoSpot) analysis (Fig. 3). While control groups showed little to no response to stimulation with either antigen, IL-17A secreting cells were found to be significantly upregulated in the PcrV-stimulated lung cells derived from the 20 μg L-PaF, 20 μg L-PaF BECC/ME and 10 μg L-PaF BECC/ME vaccinated groups (Fig. 3A). In contrast, PopB stimulation of lung cells only resulted in a significant upregulation of IL-17A for the 20 μg L-PaF BECC/ME and 10 μg L-PaF BECC/ME vaccine groups. The number of cells secreting IFN-γ following antigen stimulation was smaller than the number of IL-17A secreting cells and appeared to only be significant for 20 μg L-PaF group (Fig. 3B), suggesting that IFN-γ is less of a player for the vaccine formulations being explored here. Interestingly, unstimulated (incubated with cell culture media only) cells from the 20 μg L-PaF BECC/ME and 10 μg L-PaF BECC/ME groups showed relatively higher frequencies of IFN-γ secreting cells indicating a Th1 response upon vaccination (Fig. 3B).

Stimulation of antigen-specific mucosal immunity is an important factor for protection from mucosal pathogens^18^. Although IL-17A (and IFN-γ to a lesser extent) secreting cells were found to be present in the lungs of some vaccinated mouse groups, ELISpot does not quantify the absolute amounts of cytokines secreted by these cells. To assess this, lung cells were incubated without or with either PcrV or PopB and the cytokine levels in the culture supernatants was quantified (Supplementary Fig. 2). Both proteins stimulated lung cells from L-PaF vaccinated mice to produce significant levels of IL-17A relative to the PBS and WCK vaccinated mice. The exception was the PcrV-stimulated 10 μg L-PaF and 10 μg L-PaF with ME groups (Supplementary Fig. 2A and B). For these, IFN-γ was found to be elevated to a significant level in the 20 μg L-PaF, as expected based on the data from Fig. 3, but also in the 10 μg L-PaF BECC/ME mice (Supplmentary Fig. 2D and E). Supplementary Fig. 2C, and F shows IL-17A and IFN-γ profiles in unstimulated lung cells, respectively.

### Vaccinated mice efficiently cleared Pa from lungs

**Fig. 4:**
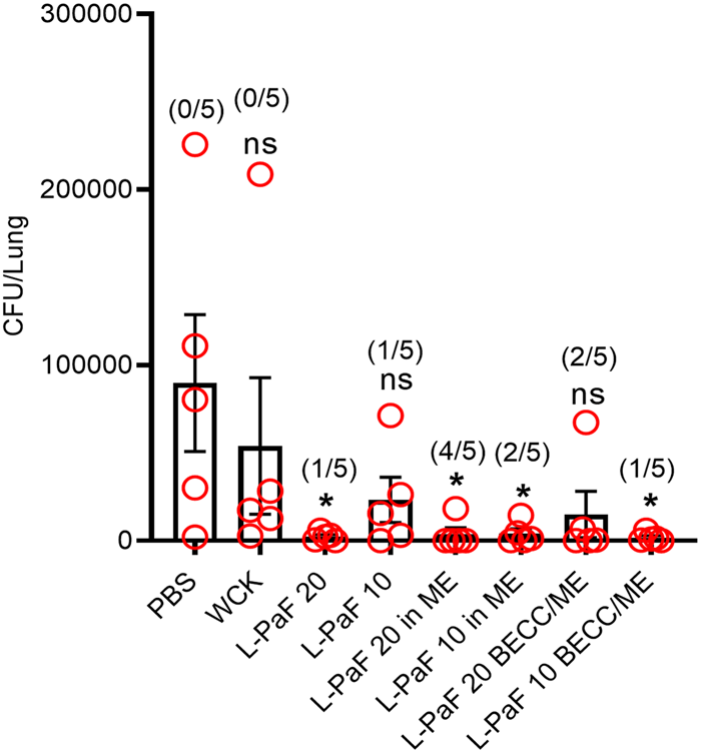
The in vivo protective efficacy of each vaccine formulation was determined. Mice were challenged with 1 × 10^8^ CFU/30 µl/mouse of mPa08-31 and lung burden was determined after 16 h post infection (HPI) (*n* = 5). The points represent individual CFU/lung values, and the SDs are denoted by error bars. Lung burden was compared between PBS and all the other groups using a Dunnett’s test. *p < 0.05.

Ultimately, rapid clearance of Pa from the lungs of challenged mice is a key hallmark of an effective vaccine. To assess this, vaccinated groups of mice were challenged with 1 × 10^8^ CFU of mPa08-31 and at 16 HPI the lungs were harvested to enumerate the relative bacterial burdens. A significant reduction of lung burden was seen for 20 μg L-PaF, 20 μg L-PaF/ME, 10 μg L-PaF/ME and 10 μg L-PaF BECC/ME vaccinated mice (Fig. 4). A noteworthy observation is that the 20 μg L-PaF BECC/ME group only failed to be statistically significant because of a single outlier. Twenty percent of mice (1/5) completely cleared Pa from the lungs of the 20 μg L-PaF, 10 μg L-PaF, and 10 μg L-PaF BECC/ME vaccinated mice. Meanwhile, 40% had completely cleared mPa08-31 in 10 μg L-PaF/ME and even the 20 μg L-PaF BECC/ME. The highest degree of complete bacterial clearance (80%) was observed for the 20 μg L-PaF/ME group. Nevertheless, the 10 μg L-PaF BECC/ME vaccinated group showed the greatest overall average reduction of Pa burden, and at a lower dose of antigen for the degree of clearance seen.

Interestingly, the CD-1 mice were also found to be protected from infection after 180 DPIm (Table 1). The results were comparable with the lung burden reductions seen in Fig. 4, however, only the 10 μg L-PaF/ME group was found to eliminate the Pa lung burden at 180 DPIm challenge. The greatest reductions in Pa burden for these mice appeared to require the presence of ME in these experiments, suggesting that a multimeric or particulate presentation of the antigen is important for long-term protection.

**Table 1:** Long term (180 DPIm) protection in CD-1 mice.

	Mean CFU/lung	% compared to PBS control
PBS	1550	100
WCK	800	51.6
L-PaF 20	140	9.0
L-PaF 10	350	22.6
L-PaF 20 in ME	125	8.1
L-PaF 10 in ME	0	0
L-PaF 20 BECC/ME	120	7.7
L-PaF 10 BECC/ME	70	4.5

Mice were challenged with 5 × 10^6^ CFU/30 µl/mouse of mPa08-31 and their lungs were harvested at 16 HPI for bacterial enumeration.

### Lung cytokine levels as related to lung bacteria burden

**Fig. 5:**
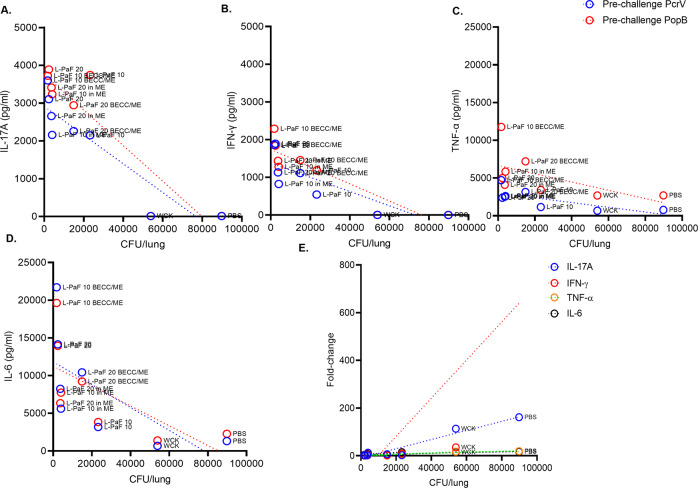
Correlation between lung burden and lung cytokines. Cytokine determination assays were performed to allow for identification of potential correlations between Pa lung burden and PcrV- or PopB-stimulated pre-challenge lung cytokines (IL-17A (**A**), IFN-γ (**B**), TNF-α (**C**) and IL-6 (**D**)). Fold changes between pre- and post-challenge untreated lung cytokines were evaluated to analyze the correlation with lung burden (**E**). Pearson’s *r* coefficient and simple linear regression (95% confidence level) were calculated. The exact values can be found in Supplementary Tables 3, and 4. The separation of points in A., B., and C. are readily seen in Supplementary Fig. 3A, B, and C, respectively.

Growing evidence suggests that a Th17 response is required for the clearance of Pa from lung^19^. Furthermore, the presence of Th1 cytokines, such as IL-6, also helps reduce Pa burden^20^. Based on these previous observations, we chose to look at markers related to a balanced Th1/Th17 response in the CD1 mouse model following IN immunization with L-PaF alone or in our formulations containing ME with or without BECC438b. IL-17A (Th17), IFN-γ, TNF-α, and IL-6 (Th1) lung cytokines were measured both pre- and post-challenge to determine if there were specific correlations with lung burden for these Th17 and Th1 markers (Fig. 5). Pre-challenge cytokines were measured upon stimulation with either PcrV or PopB, whereas post-challenge cytokines were measured in the absence of any further stimulation (as discussed above). Pre-challenge cytokines would thus mimic the effect of an actual vaccination/booster, while their post-challenge counterparts would show the cytokine profile following an in vivo infection. A negative correlation was observed between pro-inflammatory cytokines and the Pa lung burden (i.e., the higher the pro-inflammatory response pre-challenge characterized by TNF-α and IL-6, lower the lung burden is) (Fig. 5A–D and Supplementary Table 3). This observation is consistent with prior findings by others that Th1 and Th17 responses are involved in rapid pathogen clearance^16,21^. The levels of post-challenge cytokines reveal an interesting observation that a more sustained presence of controlled pro-inflammation is required as is seen by vaccination rather than the sudden onset caused by acute infection in the absence of vaccination. None of the groups vaccinated with L-PaF formulations showed a marked increase in these cytokines as a result of infection, while the PBS and WCK vaccinated groups showed a significant pro-inflammatory cytokine increase in response to the mPa08-31 infection (Fig. 5E and Supplementary Table 4). The exact fold-change values are shown in Supplementary Table 4. B.

**Fig. 6:**
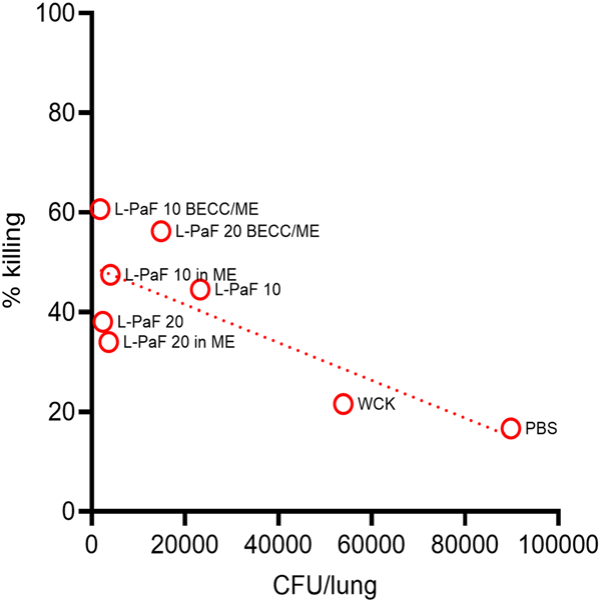
Correlation between in vitro (OPK) and in vivo reduction in lung burden. Groups with high in vitro killing ability harbor fewer lung bacteria in vivo. Pearson’s r coefficient and simple linear regression (95% confidence level) were calculated. *r* = −0.7759, 95% confidence interval = −0.9572 to −0.1572, R squared = 0.6020, *p* value (two-tailed) = 0.024.

The data shown in Fig. 5 and Supplementary Figs. 2, and 3 suggest that increased levels of pre-challenge immunoglobulins, cytokine secreting cells, and cytokines correlate with a rapid clearance of Pa for groups vaccinated with L-PaF formulations. This fits with the earlier observation that the relationship between IgG subtypes and IL-17A is an important determinant for elevated OPK activity. This is further shown as a function of serum antibodies vs. lung burden, serum IgG subtypes vs. lung burden and serum IgG subtypes vs. pre-challenge mean IL-17A (Supplementary Fig. 4A–C, respectively). Mice with higher serum antibodies showed lower lung burden (Supplementary Fig. 4A, and B, Supplementary Tables 5, and 6) and higher IL-17A (Supplementary Fig. 4C and Supplementary Table 7). OPK activity is demonstrated as one indicator of protection against Pa^22^ and, as such, it is shown to directly correlate with in vivo lung burden (i.e., higher the OPK, lower the lung burden) (Fig. 6). In line with this, the 10 μg L-PaF BECC/ME group showed highest OPK activity in vitro with the lowest overall lung burden in vivo (Fig. 6).

### Both humoral and cellular responses are important for protection: a wide range of pathways and their associated genes are activated following PcrV or PopB treatment for lung cells in vitro from vaccinated mice

Stimulation of lung cells with PcrV or PopB collected from PBS (negative control) and 10 μg L-PaF BECC/ME (the formulation showing the highest reduction of Pa lung burden) vaccinated (but not infected) mice followed by mRNA-seq analysis showed an elevated expression for genes related to the immune system (Supplementary Figs. 5, and 6). Upregulation of genes related to defense responses, innate immune responses, immune system processes, cytokines, and other effectors were observed following PcrV treatment of lung cells collected from PBS vaccinated mice. Conversely, groups vaccinated with 10 μg L-PaF BECC/ME had a slightly different gene activation pattern, most notably they had genes from the defense response, leukocyte activation, and positive responses involving different immune pathways, among other changes. Stimulation with PopB resulted in the upregulation of various other immune pathways that were not seen following PcrV stimulation (Supplementary Figs. 5, and 6). Genes from inflammatory responses, cytokine production (IL-6, IL-1β), and other immune effector genes were observed in PopB-stimulated lung cells collected from PBS vaccinated mice (Supplementary Fig. 6). Conversely, the 10 μg L-PaF BECC/ME vaccinated mice showed upregulation of genes involved in the inflammatory response pathways, immune regulation and activation, and leukocyte activation. It should be noted that, the aforementioned gene activation profiles resulted from ex vivo restimulation of uninfected but vaccinated (or PBS vaccinated negative control) lungs. When infected with Pa, they show a different activation/deactivation profile as described below.

### The lungs of 10 μg L-PaF BECC/ME vaccinated mice elicited a different gene activation profile than PBS vaccinated mice following Pa infection

**Fig. 7:**
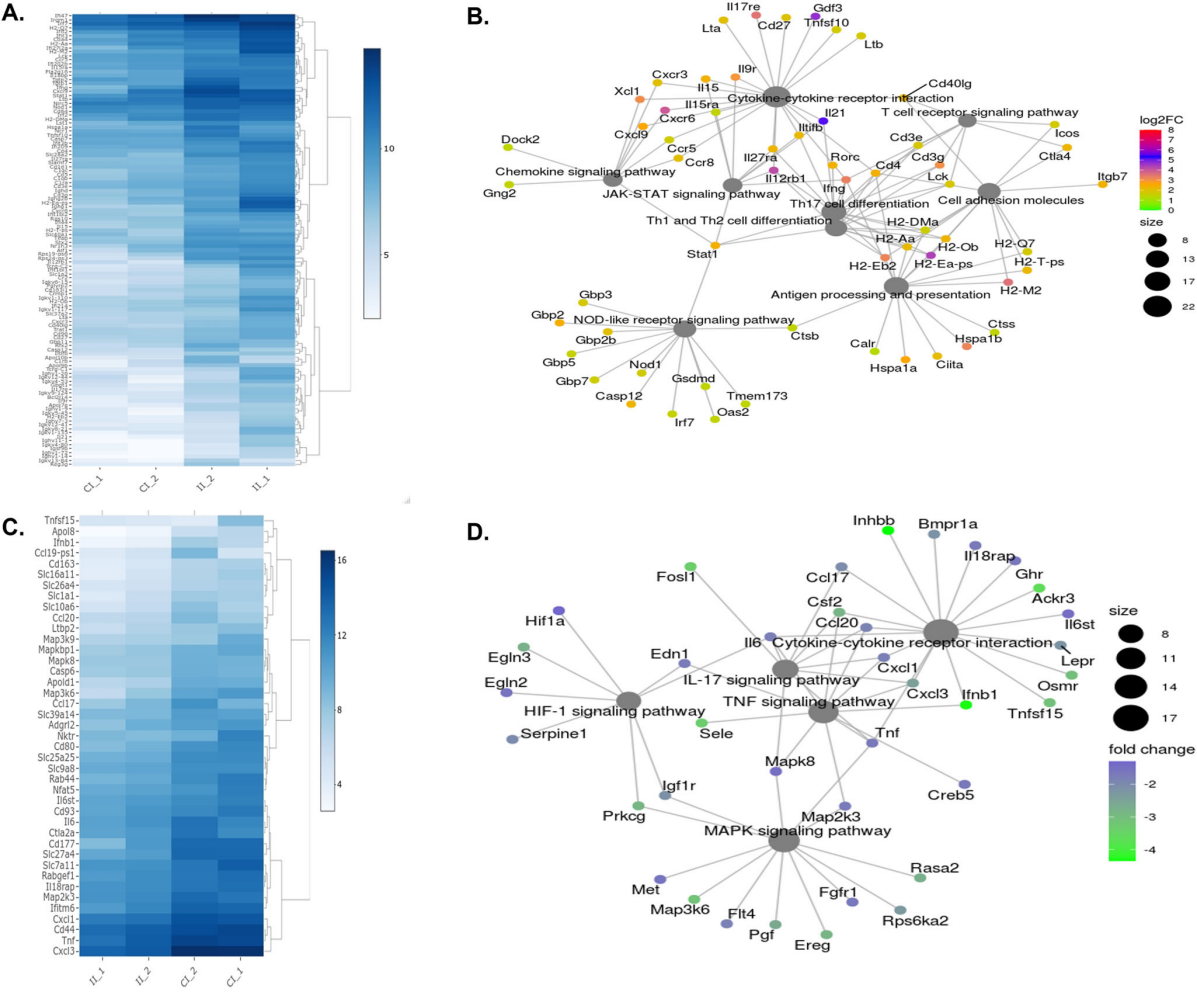
Pattern of gene up/down regulation following 16 HPI with mPa08-31 in 10 μg L-PaF BECC/ME vaccinated and PBS vaccinated mice lung cells. CI_1 = Lung cells from control or PBS vaccinated, infected group_sample #1, CI_2 = Lung cells from control or PBS vaccinated, infected group_sample #2, II_1 = Lung cells from immunized or 10 μg L-PaF BECC/ME infected group_sample #1, II_2 = Lung cells from immunized or 10 μg L-PaF BECC/ME infected group_sample #2. Upregulated genes in immunized or 10 μg L-PaF BECC/ME mice are shown in (**A**), downregulated genes in immunized or 10 μg L-PaF BECC/ME mice are shown in (**C**). **B** Pathways upregulated in infected 10 μg L-PaF BECC/ME (or downregulated in infected PBS vaccinated) mice shown via a cnet plot. Upregulated genes were found in cytokine-cytokine interaction, TCR signaling, chemokine signaling, JAK-STAT signaling, Th17, Th1/Th2 differentiation, cell adhesion molecules, antigen processing, NLR signaling pathways. **D** Pathways downregulated in infected 10 μg L-PaF BECC/ME (or upregulated in infected PBS vaccinated) mice shown via a cnet plot. Downregulated genes were found in cytokine-cytokine interaction, IL-17 signaling, TNF signaling, HIF-1 signaling and MAPK signaling pathways. Fold increase/decrease in (**B** and **D**), have been shown by different color according to the log2 scale shown in the figure. Size of the gene clusters have also been shown.

Mice vaccinated with 10 μg L-PaF BECC/ME and then challenged with Pa, showed a differential gene expression patterns relative to their PBS vaccinated counterparts to counteract as a result of the infection as determined by mRNA seq (Fig. 7A, C). Genes involved in adaptive immunity were found to be preferentially expressed in 10 μg L-PaF BECC/ME vaccinated mice, whereas genes involved with acute innate responses were preferentially expressed in the PBS vaccinated mice (Fig. 7B) with the latter leading to increased pro-inflammatory responses. These mice had experienced similar bacterial burden compared to the mice that were used for protective efficacy studies. Preferential expression of cytokine-cytokine receptor interactions, TCR signaling, chemokine signaling, JAK-STAT, Th1, Th17, cell adhesion molecules, antigen processing and presentation, and NOD-like receptor signaling pathways were observed in the lungs from the 10 μg L-PaF BECC/ME vaccinated and infected mice (Supplementary Figs. 7–12). On the other hand, the lungs of immunized mice (Fig. 7D) showed no such high expression levels for HIF-1 signaling, IL-17 signaling, TNF signaling, and MAPK (Supplementary Figs. 13–17). The differential expression of these genes is shown with graded red and green colors, respectively. Genes that were preferentially expressed in 10 μg L-PaF BECC/ME mice were downregulated, relatively speaking, in PBS-vaccinated mice and vice-versa. Notably, the highly expressed HIF-1 signaling in infected PBS-vaccinated mice suggests that lung damage is occurring, and there is an associated pro-inflammatory storm, that leads to higher bacterial burden in the lungs^23,24^.

### IL-17 and immunoglobulins are important determinants of protection

**Fig. 8:**
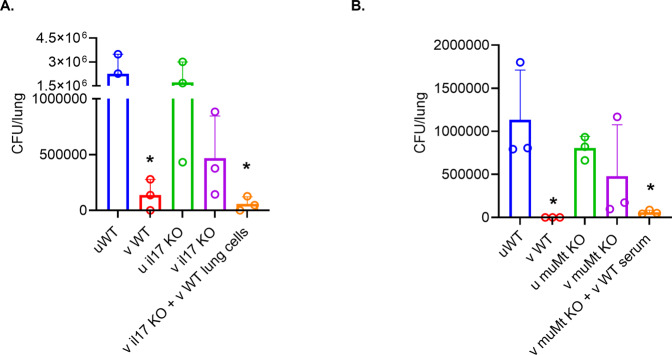
In vivo protective efficacy study in knock out (KO) mice. **A** IL-17 KO or (**B**) muMt^-^ KO mice were infected with 1 × 10^8^ CFU/30 µl/mouse and their lungs processed at 16 HPI to evaluate lung bacterial burden (*n* = 3). The points represent individual CFU/lung values, and the SDs are denoted by error bars. Lung burden was compared between uWT and other groups using Dunnett’s test. *p < 0.05, **p < 0.01, ***p < 0.001. uWT = PBS vaccinated wild type; vWT = 10 μg L-PaF BECC/ME vaccinated WT; u il17 KO = PBS vaccinated IL-17 K; v il17 KO = 10 μg L-PaF BECC/ME vaccinated IL-17 KO; u muMt KO = PBS vaccinated muMt KO; v muMt KO = 10 μg L-PaF BECC/ME vaccinated muMt KO mice.

Infection with mPa08-31 was successfully hindered by 10 μg L-PaF BECC/ME (“v” groups) vaccination in the WT group (Fig. 8). Both PBS (“u” groups) and 10 μg L-PaF BECC/ME vaccinated *il17*^*−/−*^ groups showed a high lung burden (Fig. 8A). When the 10 μg L-PaF BECC/ME vaccinated *il17*^*−/*−^ mice were complemented with lung cells from 10 μg L-PaF BECC/ME vaccinated WT, the infection was significantly reduced. Administration of rat anti-mouse IL-17A (IL-17A Rat anti-Mouse, NA/LE, Unlabeled, clone: TC11-18H10, catalogue #: BDB560268, 1:50 dilution) antibody does not dampen the host immune response. High levels of immunoglobulins and in vivo IL-17A were observed in these mice (Supplementary Figs. 18, and 19). High levels of IL-17A post-challenge correlated with lower bacterial burden (Supplementary Fig. 19A, and C), whereas higher IL-6 correlated with higher lung burden (Supplementary Fig. 19B, and D).

Colonization with mPa08-31 was hindered in 10 μg L-PaF BECC/ME vaccinated WT mice, as expected. Conversely, PBS and 10 μg L-PaF BECC/ME vaccinated muMt^-^ KO mice had higher bacterial burdens in their lung compared to the 10 μg L-PaF BECC/ME vaccinated WT group (Fig. 8B). Although not statistically significant, a clear difference was observed in the bacterial burden between PBS and 10 μg L-PaF BECC/ME vaccinated muMt^-^ KO groups. Upon receiving serum from 10 μg L-PaF BECC/ME vaccinated WT mice, lung burden in the 10 μg L-PaF BECC/ME vaccinated muMt^-^ KO mice were found to be comparable with 10 μg L-PaF BECC/ME vaccinated WT group. Addition of IL-17 neutralizing antibody showed no effect on 10 μg L-PaF BECC/ME vaccinated muMt^-^ KO mice. As expected, the muMt^-^ KO mice could not produce any immunoglobulins (Supplementary Fig. 20). They did produce IL-17A and IL-6, as well as other cytokines, leading to a scrambled immune response (with no humoral response and somewhat activated cellular response) (Supplementary Fig. 21A, and B). Although not statistically significant, a trend was observed when lung IL-17A and IL-6 post-challenge was compared with the lung burden with a visual correlation observed with higher IL-17A leading to a better protection (Supplementary Fig. 21C, and D).

## Discussion

Pa is a ubiquitous opportunistic pathogen that is an important cause of nosocomial infections in immune-compromised individuals, burn and wound victims, patients with cystic fibrosis, and the elderly. A licensed vaccine remains an unmet need. Different approaches have been devised to construct a vaccine against Pa, but with limited success thus far^8–12^. Recently, our group has assessed the efficacy of a new T3SS subunit vaccine using a mouse acute lung model^13^. The T3SS is an important virulence factor involved in initiating Pa infection. The Pa T3SS injects effectors into host cells, impairing early innate immune responses to allow initiation of infection^25^, but this requires a functional secretion apparatus (T3SA) with functional tip complex and translocator proteins. Since the T3SS is employed in the early stages of infection, proteins associated with T3SA can potentially be important immune targets for preventing the onset of infection^26^.

In this study, the T3SA tip and first translocator proteins, PcrV and PopB, respectively, were genetically fused with *E. coli* LTA1 to produce L-PaF. Mice were vaccinated with two concentrations of L-PaF with or without other adjuvants, i.e., MedImmune Emulsion (ME) and BECC438b (BECC). ME is an oil-in-water emulsion that we use to create a nanoparticle that enhances multimeric presentation of the antigen to increase the resulting immune response^17,27^. While monomeric protein antigens elicit robust immune responses and are often efficacious in mice, they often fail in human trials unless formulated into an oligomeric presentation with an appropriate adjuvant^28,29^. BECC438b is a TRL4 agonist that is a biosimilar of monophosphoryl lipid A (MPLA)^30^. Immunogenicity of L-PaF was found to increase with the addition of ME and BECC. Serum immunoglobulins were elevated at 42 DPIm, with a small decrease on 56 DPIm. Serum from mice vaccinated with L-PaF formulations showed enhanced killing capacity for wild-type Pa in in vitro OPK studies. This bacterial killing ability was further enhanced for formulations involving ME and BECC. Additionally, OPK activity had a positive correlation with serum IgG, IgA, and certain IgG subtypes.

Pa has been reported to be subjected to killing in the presence of a balanced Th1/Th2 antibody and opsonophagocytic response^13,31,32^. A marker for a balanced Th1/Th2 response is a balanced IgG1/IgG2a ratio^33^. Membrane proteins and soluble protein antigens usually upregulate IgG1, while responses against capsular polysaccharide are restricted to IgG2. On the other hand, IgG3 is a potent inflammatory antibody with comparatively shorter half-life but with the ability to effectively direct OPK^34^. In our study, a steady increase was observed for IgG1, IgG2a and IgG3 following vaccination. Studies have uncovered the ability of different IgG subtypes to bind with components of the immune system, such as binding with FcγRs and complement C1q components, to name a few^35^. A steady presence of different immunoglobulins, as well as their involvement with OPK activity, provides a primary indication that our L-PaF formulations are indeed steering the host immune response in a desirable direction.

Many bacterial infections are dependent on the host’s immune status. A Th2-dominant pulmonary response in mice is seen to be susceptible to Pa infection. Meanwhile, a Th1-dominant response has a better prognosis against Pa^36^. Involvement of a Th17 cell response in the form of IL-17 has also been shown useful for Pa clearance^13,16,21^. Secretion of the Th1 cytokine IFN-γ, albeit modestly, and IL-17 in the present study suggest a favorable outcome post infection. Indeed, reduced lung Pa burden at 16 HPI shows a clear correlation with the addition of the adjuvants used here. More importantly, this protective efficacy shows a dose-dependent response with the presence of ME and BECC. Mice were further checked for long-term protective efficacy at 6 months post first vaccination with significant success for the formulated antigen.

Th1-Th17 response plays an important role in determining the fate of an infection. Differentiation of naïve T cells into Th1/Th2 and/or Th17 depends on direct interaction with APCs, cytokine environment and coreceptors signals, among others^37^. In our study, a clear Th1 differentiation was observed in vaccinated and infected mouse lungs (Supplementary Fig. 9). We hypothesize this effect is elicited by the BECC438b adjuvant (a TLR4 agonist) in conjunction with the multimeric presentation of the antigens. Historically, LPS has been associated with eliciting a Th1-biased immune response^38^ and our study would agree with those findings. Activation of various upstream TCR signaling pathways (Supplementary Fig. 8) leading to the production of IFN-γ, as well as STAT1 and IL-12, in mice vaccinated with the L-PaF formulation, but not in PBS vaccinated mice, is an indication that Pa infection proceeds differently in the vaccinated mice. Moreover, upregulation of RORγt, IL-21 and IL-22 indicates a pronounced Th17 immune response following infection in vaccinated mice. IL-21 and IL-22 are known to mount an immune response against extracellular pathogens and play a protective role in mucosal anti-microbial host defenses^39^. While a protective immune response was observed in mice vaccinated with the L-PaF formulation, a more pronounced pro-inflammatory response was observed in PBS vaccinated mice subsequently infected with Pa. Genes from TNF, MAPK and other pro-inflammatory cytokines were downregulated in mice vaccinated with the L-PaF formulation, while these genes were upregulated, leading an uncontrolled inflammatory response, in PBS vaccinated and subsequently infected mice. Hypoxia inducible genes were also found to be upregulated in PBS vaccinated mice after infection. Hypoxia is a factor known to activate NF-κB, leading to an uncontrolled release of pro-inflammatory cytokines, such as IL-6, TNF-α and MCP-1, to name a few^40^. Downregulation of TNF, IL-17 and MAPK downstream pathways following infection in mice vaccinated with the L-PaF formulation, but not in PBS vaccinated mice indicates that PBS vaccinated mice reacted severely to the infection, while the mice vaccinated with the L-PaF formulation showed a more protective effect after infection. PBS vaccinated mice tried to circumvent the infection by activating their innate response gene manifold, while mice vaccinated with the L-PaF formulation were more “prepared” for the infection and eliminated it more rapidly.

Although several novel pathways were found to provide the observed anti-Pa immunity that have not been shown before, it was not possible to assess all of them within the scope of the present work. A controlled IL-17 response, along with serum immunoglobulins, were found to be associated with anti-Pa immunity in our previous studies, as well^17^. Thus, we chose these two main protective immune components to assess their effects in specific KO mice. In both cases, a dampened anti-Pa response suggests that both IL-17 and immunoglobulins are needed to generate a protective anti-Pa immunity in mice.

Additional mutational and molecular approaches are needed to fully understand the immune pathways and responses needed to mount effective anti-Pa immunity. Nevertheless, this work provides a preliminary view of the basic pathways required to generate an anti-Pa immunity and to eliminate the infection within the context of the formulations used here. How alternative formulations might affect the efficacy and immune pathways elicited remains to be determined. Future work can build upon the present work as a knowledge base for generating new information.

## Methods

### Materials

Squalene was purchased from Echelon Biosciences (Salt Lake City, UT). BECC438b was extracted from *Yersinia pestis* after the introduction of lipid A modifying enzymes. It is a bis-phosphorylated lipid A analogue generated using a Bacterial Enzymatic Combinatorial Chemistry (BECC) platform in the *Yersinia pestis* KIM6 strain^30^. Candidate number 438 was given the designation BECC438b to indicate that it is purified from a biological (b) origin. The BECC438b used here has been tested and found to be nontoxic in rabbits. All other buffers, and chemicals were reagent grade.

### Proteins and formulations

L-PaF was purified using standard IMAC and Q anion exchange column chromatography steps^13,17^. L-PaF was dialyzed into PBS with 0.05% LDAO and stored at −80 °C. The formulations were prepared as oil-in-water emulsions^13,17^. Briefly, squalene (8% by weight) and polysorbate 80 (2% by weight) were mixed to achieve a homogenous oil phase. Twenty percent sucrose and 40 mM histidine (pH 6) were added to the oil phase to generate a milky emulsion of 4XME (MedImmune Emulsion). BECC438b (2 mg/ml) was prepared in 0.5% triethanolamine and adjusted to pH 7.2 with 1 M HCl. To make the L-PaF with ME formulation, the protein was added directly to the ME with a final concentration of 0.67 mg/ml. To make the L-PaF with ME and BECC438b formulation, ME and BECC438b were first mixed and then L-PaF was mixed with ME-BECC438b solution at a volumetric ratio of 1:1 to achieve the desired final antigen concentration.

### Mice and immunizations

Six- to eight-week-old CD-1, C57BL/6 (B6), B6.Cg-*Il17a/Il17f*^*tm1.1Impr*^ *Thy1*^*a*^/J (*il17*^−*/−*^ or IL-17 KO) and B6.129S2-*Ighm*^*tm1Cgn*^/J (muMt^−^ KO) mice were purchased from Charles River Laboratories (Wilmington, MA) or The Jackson Laboratory (ME, USA). Mice were left to acclimate for 1 week following their delivery. Eight groups of CD-1 mice (*n* = 10/group) were vaccinated intranasal (IN) with 30 µl containing PBS, Whole Cell Killed (WCK) Pa, 20 μg L-PaF (20 μg L-PaF), 10 μg L-PaF (10 μg L-PaF), 20 μg L-PaF in ME (20 μg L-PaF/ME), 10 μg L-PaF in ME (10 μg L-PaF/ME), 20 μg L-PaF+BECC438b in ME (20 μg L-PaF BECC/ME), and 10 μg L-PaF+BECC438b in ME (10 μg L-PaF BECC/ME). Formulations were administered in a prime-boost-boost manner with the prime dose on day 0, followed by boosters on days 14 and 28. Blood was collected on days 0, 28, 42 and 56. *il17*^*−/*−^, muMt^-^ and C57BL/6 were only vaccinated with PBS or the 10 μg L-PaF BECC/ME formulation and assessed for protection since this formulation was found to be protective in a preliminary trial (*n* = 3).

### Ethical statement

Animal works were carried out according to the University of Kansas (KU, Lawrence) IACUC animal use statement (AUS 222-03, valid until March 9, 2025). The Institution’s Animal Welfare Assurance number is D16-00220 (A3339-01).

### ELISA

Serum anti-PcrV, anti-PopB IgG and IgA were measured by coating the 96-well microtiter plates with 100 µl of either PcrV (1 µg/ml) or PopB (1 µg/ml) and incubated for 3 h^41^. Plates were then blocked with 10% non-fat dry milk overnight at 4 °C. After washing, primary (serum) and secondary antibodies (1:1000 dilution for anti-IgG, and 1:4000 for anti-IgA), [Catalogue #: OB1040-05 for IgA from Southern Biotech, and catalogue #: 5450-0011 (474-1806) for IgG from Sera Care] were added and incubated for 1 h each followed by washes with PBS-Tween 20 (5%). All secondary antibodies were horseradish peroxidase (HRP)-labeled goat anti-mouse IgG/IgA, that were human-adsorbed. TMB substrate (3, 3′, 5, 5′-tetramethylenebenzidine) was added and the reaction was stopped with phosphoric acid. Endpoint titers were calculated as ELISA units per ml (EU/ml). IgG1, IgG2a and IgG3 subclasses were measured similarly with the appropriate secondary antibody.

### Opsonophagocytic killing (OPK) assay

OPK assay was carried out using the Pa strain mPa08-31 that was grown overnight^13^. The following morning, a new culture was started using 1:100 inoculum in low salt LB broth (0.5% w/v NaCl) and the absorbance (600 nm) was adjusted to 0.3. A bacterial concentration of 2 × 10^7^ cells/ml was adjusted in 10% bovine serum albumin (BSA, Sigma, St. Louis, MO) containing Minimal Essential Medium (MEM, ThermoFisher, Waltham, MA). The J774.1 macrophage cell line (ATCC, Manassas, MA) was grown in Dulbecco’s Modified Eagle’s Medium (DMEM, ThermoFisher, Waltham, MA) until 90% confluence was achieved. mPa08-31 was adjusted to a final Multiplicity of Infection (MOI) of 0.1. Sera from each mouse group at 42 DPIm were heat inactivated at 56 °C for 30 min. The serum was diluted 1:500 and then mixed with the bacteria and macrophages at a ratio of 1:1:1 to a final volume of 300 µl. This mixture was incubated for 30 min at 37 °C and serial dilutions were prepared for all the technical quadruples and plated on PIA. Percent killing was measured using the following formula:

$$\left[ {\left\{ {\left( {{{{\mathrm{CFU}}}}\,{{{\mathrm{from}}}}\,{{{\mathrm{T}}}}_0} \right) - \left( {{{{\mathrm{CFU}}}}\,{{{\mathrm{from}}}}\,{{{\mathrm{T}}}}_{30}} \right)} \right\}/\left( {{{{\mathrm{CFU}}}}\,{{{\mathrm{from}}}}\,{{{\mathrm{T}}}}_0} \right) \times 100} \right].$$

### Adoptive transfer

Adoptive transfer of lung cells and serum from vaccinated mice was done for *il17*^*−/−*^ and muMt^−^ mice, respectively. Lungs were collected from B6 mice vaccinated with 10 μg L-PaF BECC/ME and a single cell suspension (1 × 10^6^ lung cells/ml) was prepared. Each mouse received 100 µl (1 × 10^5^ lung cells) 3 h prior to the bacterial challenge. Serum was collected from B6 mice vaccinated with 10 μg L-PaF BECC/ME as described above. Serum (100 µl) was transferred to each of the recipient mice. Both cells and serum were introduced intraperitoneally^42^.

### Bacterial challenge experiment

The Pa strain mPa08-31^43,44^ was grown overnight and used to inoculate fresh low salt LB medium at a 1:100 ratio. The bacterial culture was incubated at 37 °C until it reached an OD_600_ of 0.3. Pa was then centrifuged and adjusted to a concentration of 1 × 10^8^ CFU/30 µl. Two months after the first immunization, or 4 weeks after the last booster, mice were anesthetized using isoflurane and challenged intranasally (IN) with 30 µl of the bacterial suspension. The waiting period of 4 weeks ensures that the innate response has subsided and that any observed protection is from adaptive immunity. At 16 HPI, mice were euthanized, and the organs were processed as described below. The lung cells were then used to evaluate bacterial burden. The same bacterial concentration was used for the CD-1, B6 and the different KO mice. For mRNA seq. of the infected mice, a separate experiment was carried out (*n* = 2/group) with two groups. The groups used here were the PBS immunized or control and L-PaF 10 BECC/ME immunized or immunized. They were challenged at the same dose of Pa, and RNA was isolated at 16 HPI. For long-term protective efficacy, mice were challenged with 5 × 10^6^ CFU bacterial inoculum per mouse at 180 DPIm. Lungs were assessed at 16 HPI for the analysis of lung burden as described above.

### Organ collection

For immunological analysis of the lung, they were collected aseptically in MACS^®^ Tissue Storage Solution (Miltenyi Biotec, USA) and processed with a lung dissociation kit (Miltenyi Biotec, USA). An erythrocyte lysis step was carried out followed by adjusting the cell number to 1 × 10^7^ cells/ml. These cells were then further processed for cytokine and ELISpot studies. Pre-challenge necropsies were carried out at 56 DPIm, while post-challenge necropsies were carried out at 16 HPI.

### T cell ELISpot assay

Lung cells from the previous step were incubated for 24 h at 37 °C in the presence or absence of 5 µg/ml of either PcrV or PopB. ELISpot plates were coated with capture antibodies against IFN-γ or IL-17A for a T cell double-color enzymatic assay according to the manufacturer’s instructions (ImmunoSpot, Cellular Technology Limited, USA). Cytokine secreting cells were quantified using an ImmunoSpot analyzer with Professional DC software. A quality control step was carried out before finalizing the data.

### Cytokine analysis

Lung cells were incubated for 48 h at 37 °C in the presence or absence of 10 µg/ml of either PcrV or PopB. Supernatants were analyzed with U-PLEX kits for the following cytokines: IFN-γ, IL-17A, IL-6 and TNF-α. Cytokine concentrations were measured using a Meso Scale Discovery (MSD, Rockville, MD) plate reader using DISCOVERY WORKBENCH^®^ analytical software. Out of the 10 cytokines measured, only IL-17A, IFN-γ (pre-challenge) and IL-17A, IFN-γ and TNF-α (post-challenge) levels were found to be significantly changed when compared to their respective control groups.

### Correlation studies

Linear correlation studies were carried out using bivariate correlation in the form of Pearson r. They were further analyzed via simple linear regression. P value designations are discussed in the statistical analysis section. All fold change studies were done using the pre-challenge unstimulated lung cytokines as the denominator.

i. OPK and immunoglobulins. The day 42 serum samples’ in vitro bacterial killing ability and serum immunoglobulin responses were analyzed for linear correlation using the methods described above. OPK was also analyzed against IgG subtypes.
ii. Cytokines and lung burden. Pre- and post-challenge lung cytokines and lung burden were analyzed for linear correlation as described above.
iii. OPK and lung burden. The in vitro bacterial killing ability of serum samples was cross-checked with in vivo lung burden to determine whether there is a possible correlation between the two.
iv. IgG subtypes and IL-17A. Serum IgG subtypes were cross-checked with the protective cytokine IL-17A to determine whether there is a correlation between them.
v. IgG subclasses and lung burden. IgG subclasses were also analyzed to determine whether there is any correlation between these with the in vivo lung burden.

### mRNA-seq analysis

RNA was isolated from lung cells using RNeasy® Mini kit according to the manufacturer’s instructions (QIAGEN, Hilden, Germany). RNAs with an RNA Integration Number (RIN) of >7 were shipped to Novogene for mRNA sequencing. Another round of QC was carried at their facility prior to the actual mRNA sequencing. mRNA-seq read count data was provided by Novogene which is further processed using the iDEP web server (PMID: 30567491) for differential expression and pathway analysis. Initially, only the genes with at least 0.5 counts per million (CPM) reads in at least one sample were considered. Read counts were then transformed as log2(CPM) using the EdgeR method. The differentially expressed genes (DEGs) were identified using DESeq2 with an FDR cutoff 0.05 and a minimum fold change of 2. Finally, the enriched pathways in DEGs for the selected comparisons were identified through GO Biological Process analysis. Up-regulated pathways which most likely play a critical role in immune modulation were manually selected for each comparison. The fold change of individual genes associated with selected pathways were visualized as a heatmap using R. Gene Set Enrichment Analysis of Gene Ontology and KEGG pathway analysis was performed using R clusterProfiler (version 3.0.4).

### Statistical analyses

GraphPad Prism 8.1.2 was used to prepare data and perform statistical analyses. PBS vaccinated groups were compared with the other vaccinated groups using Dunnett’s multiple comparison test. A *p* value of <0.05 was considered significant (*p < 0.05, **p < 0.01, ***p < 0.001). Pearson’s *r* values and R squared values are mentioned as deemed appropriate. mRNA sequencing data were prepared as stated above.

### Reporting summary

Further information on research design is available in the Nature Research Reporting Summary linked to this article.

## Supplementary information

Supplemental Materials.

REPORTING SUMMARY

## Data Availability

Data will be made available upon request.
