# Supplementary material for: A protein subunit vaccine elicits a balanced immune response that protects against Pseudomonas pulmonary infection
Source: NPJ Vaccines. 2023 Mar 14;8:37. doi: 10.1038/s41541-023-00618-w (PMC10012293; doi:10.1038/s41541-023-00618-w)
Supplement: Supplementary file 1 — Supplemental Materials. [file 41541_2023_618_MOESM1_ESM.pdf]

SUPPLEMENTARY MATERIALS FOR

A protein subunit vaccine elicits a balanced immune response that protects against *Pseudomonas* pulmonary infection

Debaki R. Howlader[#1,4], Sayan Das[#3], Ti Lu[1, 4], Rahul Shubhra Mandal[2], Gang Hu[1], David J. Varisco[3], Zackary K. Dietz[1,4], Siva Sai Kumar Ratnakaram[1], Robert K. Ernst[3], William D. Picking[1,4], and Wendy L. Picking[1,4*]

[1]Department of Pharmaceutical Chemistry, University of Kansas, Lawrence, Kansas 66047, [2]Perelman School of Medicine, University of Pennsylvania, Philadelphia, PA, 19104, [3]Department of Microbial Pathogenesis, University of Maryland, Baltimore, MD 21201

[4]Current address: Department of Veterinary Pathobiology, University of Missouri, Columbia, MO 65211

*Corresponding Author:

Wendy L Picking, wendy.picking@ku.edu

# These authors share equal authorship.

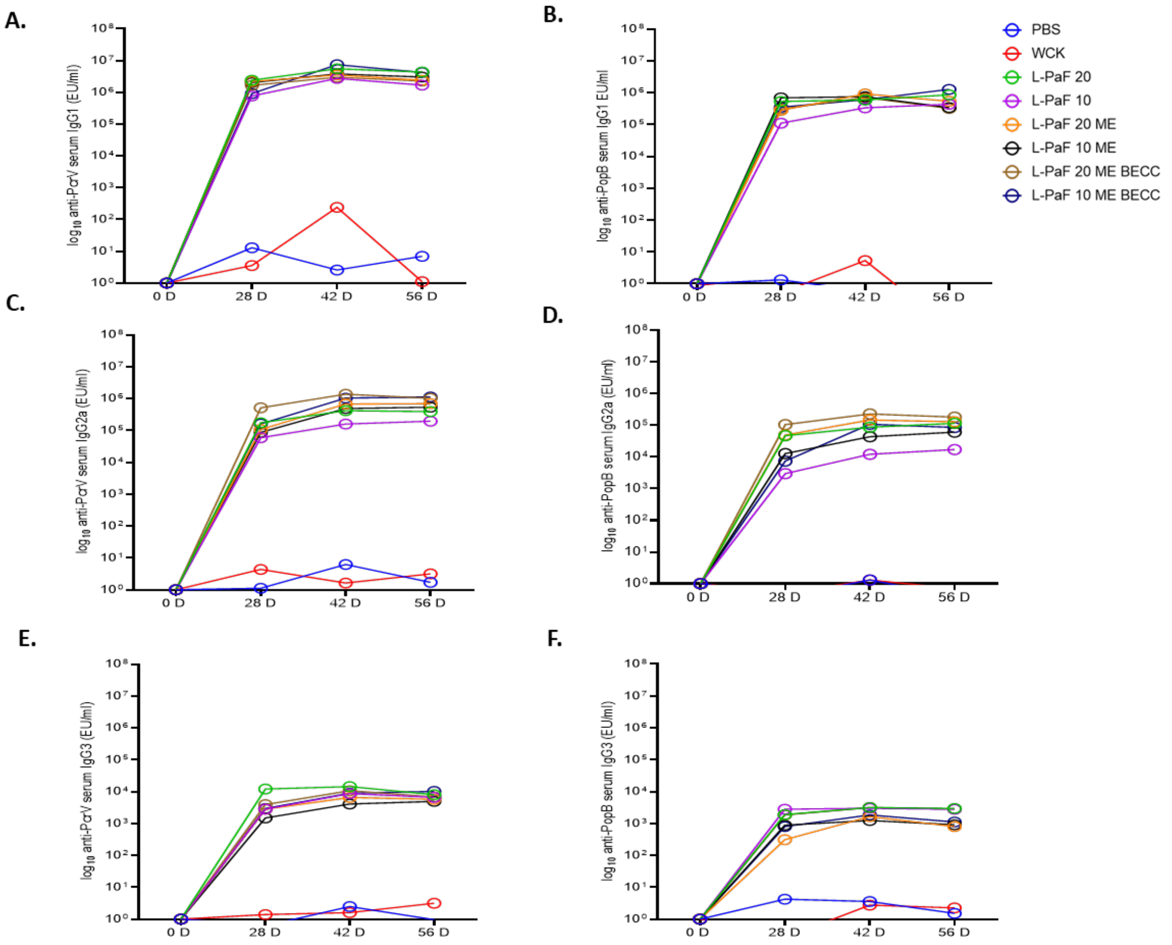

Supplementary Figure 1. Kinetics of serum IgG subtypes. CD-1 mice were vaccinated on days 0, 14 and 28 and their sera were assessed for anti-PcrV and anti-PopB IgG subtype immunoglobulins as a function of time and boosting. Anti-PcrV IgG1 **(A),** IgG2a **(C)**, and IgG3 **(E)**; along with anti-PopB IgG1 **(B)**, IgG2a **(D)**, and IgG3 **(F)** are shown. Pooled titers are represented as EU/ml. Each point denotes a value of the pooled titer (n = 10 mice/group).

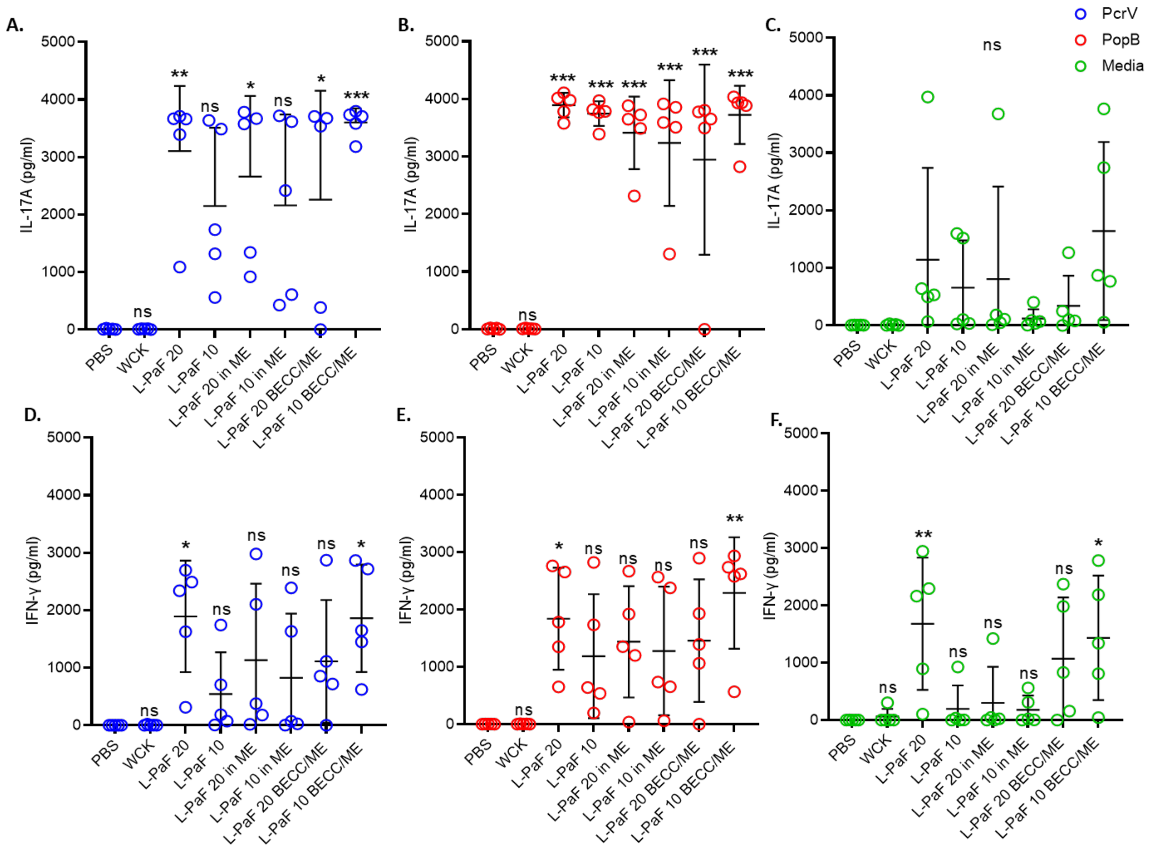

Supplementary Figure 2. Secretion of IL-17A or IFN-γ from stimulated lung cells pre-challenge. Lung cell suspensions were prepared and treated with PcrV, PopB or media for 48 hours at 37°C. Secretion of IL-17A was measured in **(A)** PcrV, **(B)** PopB and **(C)** media treated cells by MesoScale Discovery (MSD) as per the manufacturer's instructions. Secretion of IFN-γ was measured in **(D)** PcrV, **(E)** PopB and **(F)** media treated cells by the same procedure. Each dot represents actual values and error bars shows SD (n = 5/group). The values were compared with PBS using a two-way ANOVA (Dunnett's test). *p < 0.05, **p < 0.01, ***p < 0.001.

A.

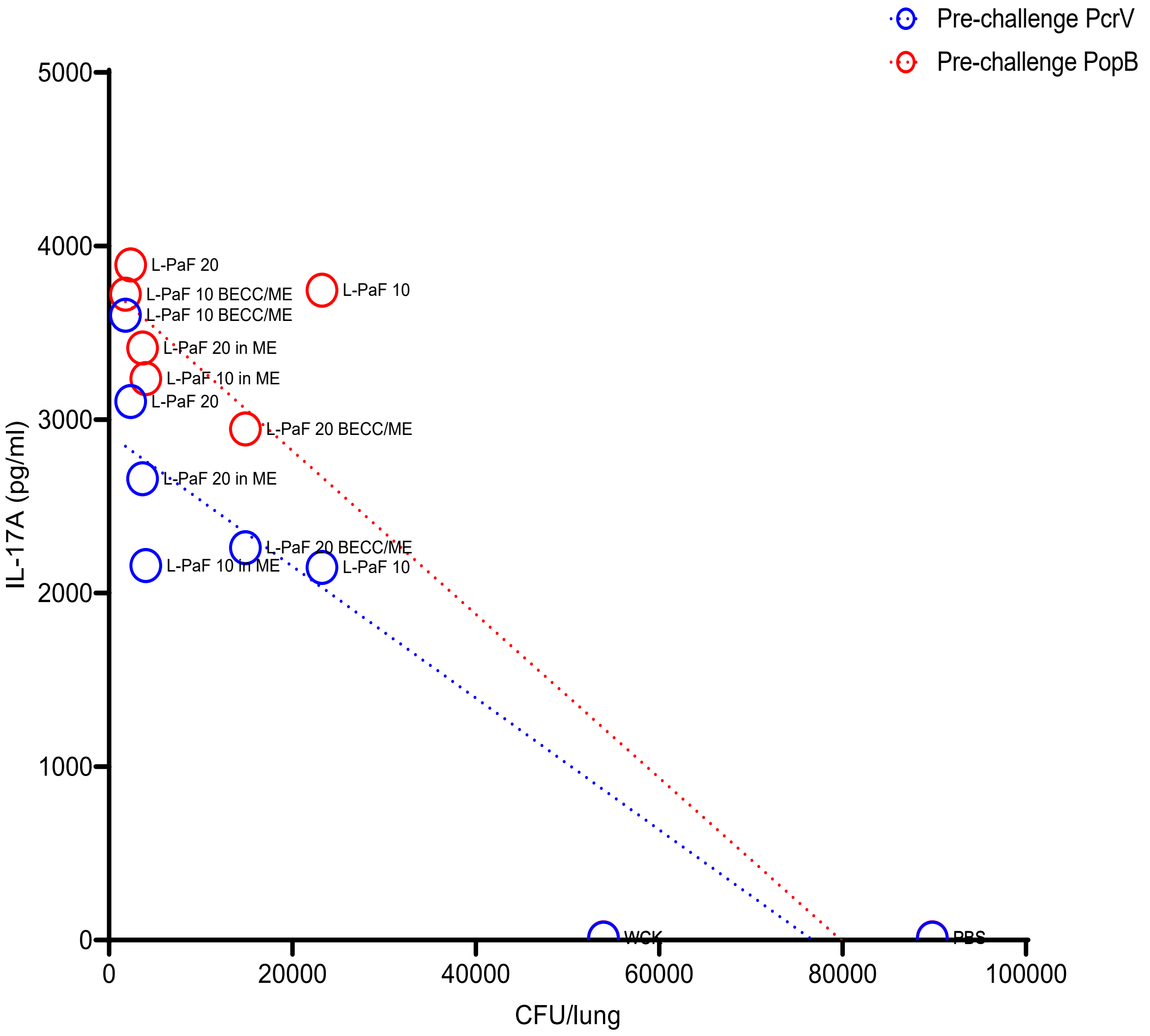
Pre-challenge PcrV
Pre-challenge PopB
IL-17A (pg/ml)
CFU/lung
L-PaF 20
L-PaF 10 BECC/ME
L-PaF 10
L-PaF 10 BECC/ME
L-PaF 20 in ME
L-PaF 10 in ME
L-PaF 20
L-PaF 20 BECC/ME
L-PaF 20 in ME
L-PaF 20 BECC/ME
L-PaF 10 in ME
L-PaF 10
WCK
PBS
0
1000
2000
3000
4000
5000
0
20000
40000
60000
80000
100000

B.

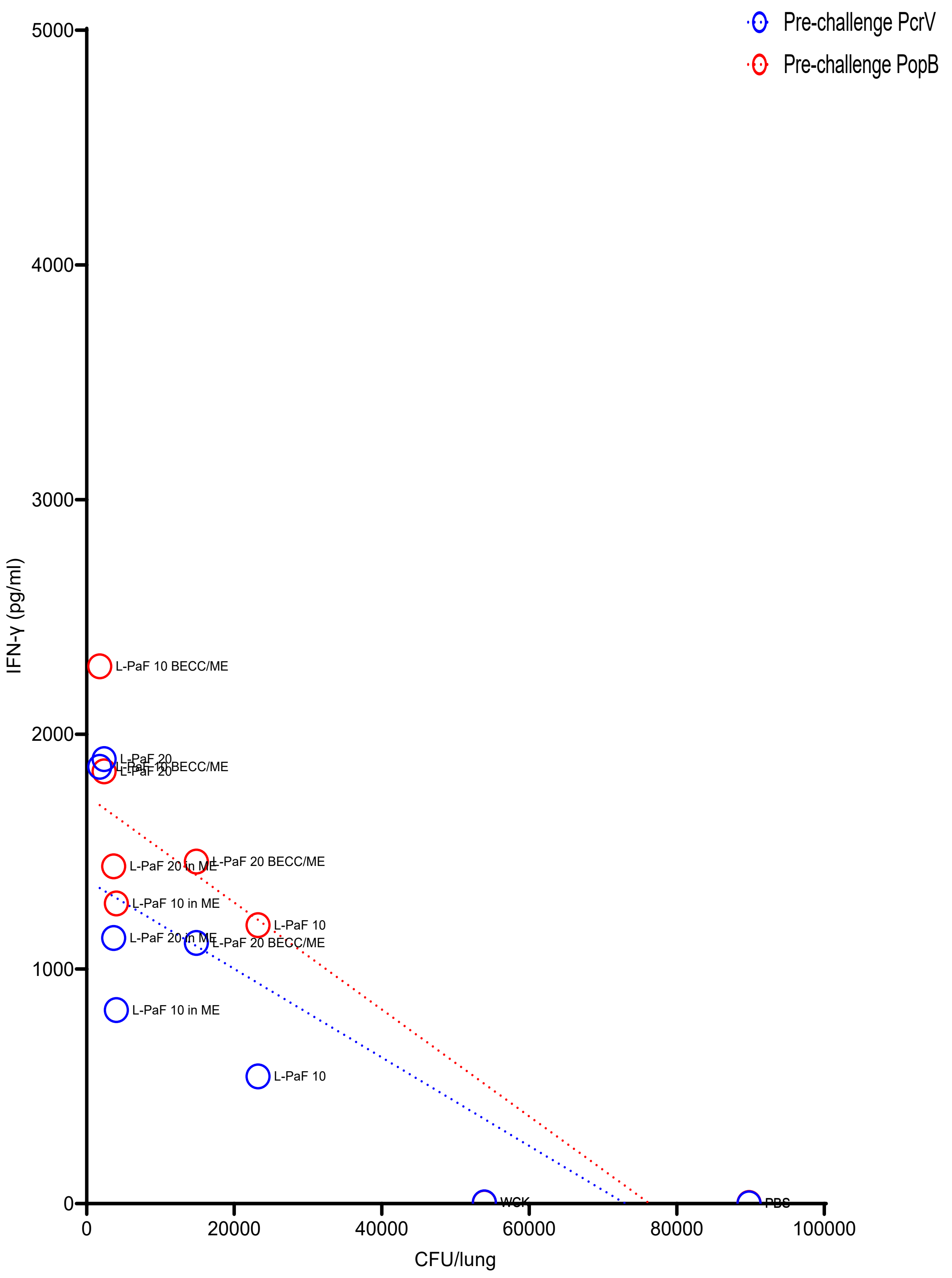

Pre-challenge PcrV
Pre-challenge PopB
IFN-γ (pg/ml)
5000
4000
3000
2000
1000
0
0
20000
40000
60000
80000
100000
CFU/lung
L-PaF 10 BECC/ME
L-PaF 20
L-PaF 10 BECC/ME
L-PaF 20
L-PaF 20 in ME
L-PaF 20 BECC/ME
L-PaF 10 in ME
L-PaF 10
L-PaF 20 in ME
L-PaF 20 BECC/ME
L-PaF 10 in ME
L-PaF 10
WCK
PBS

C.

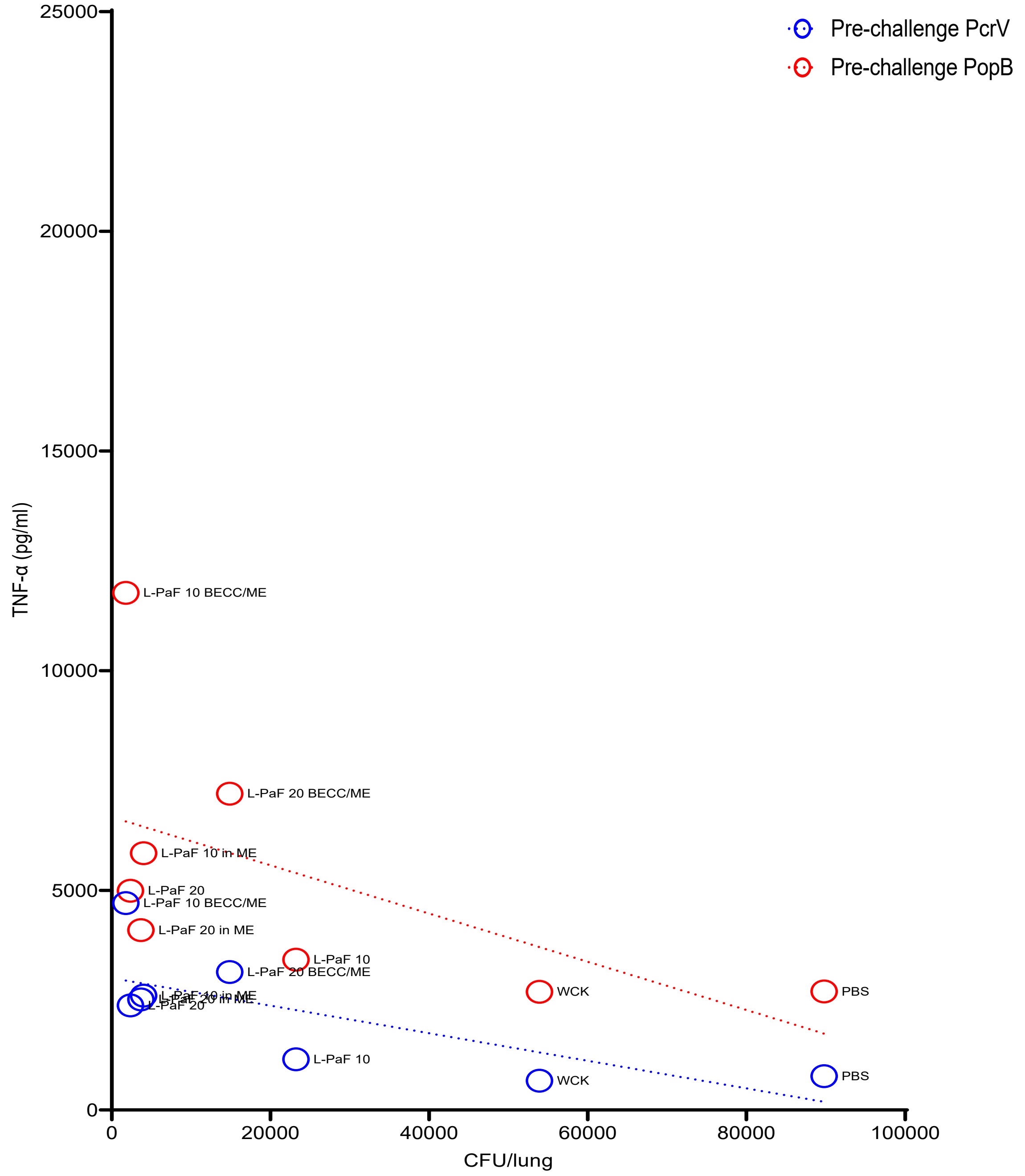

Supplementary Figure 3. Stretched out version of Figure 5.A., B., and C. Please see text for details.

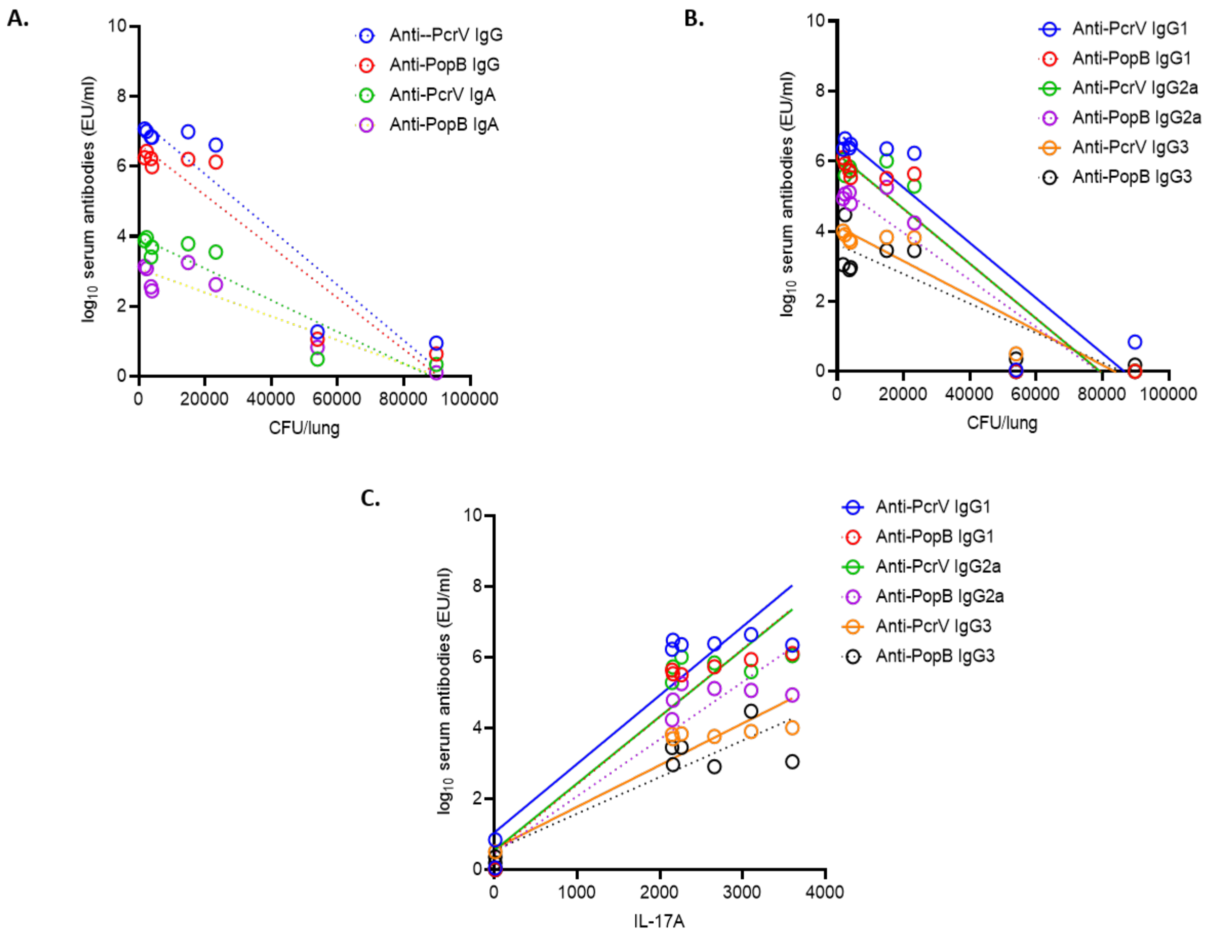

Supplementary Figure 4. Correlation between lung burden, serum immunoglobulins and lung IL-17A. Correlation assays were performed to identify correlations between **(A)** lung burden and anti-PcrV, anti-PopB IgG, IgA or **(B)** lung burden and serum IgG subtypes of against PcrV and PopB. Correlation assay was also performed between **(C)** post-challenge IL-17A from media treated lung versus different subtypes of serum IgG against PcrV and PopB Pearson's r coefficient and simple linear regression (95% confidence level) were calculated.

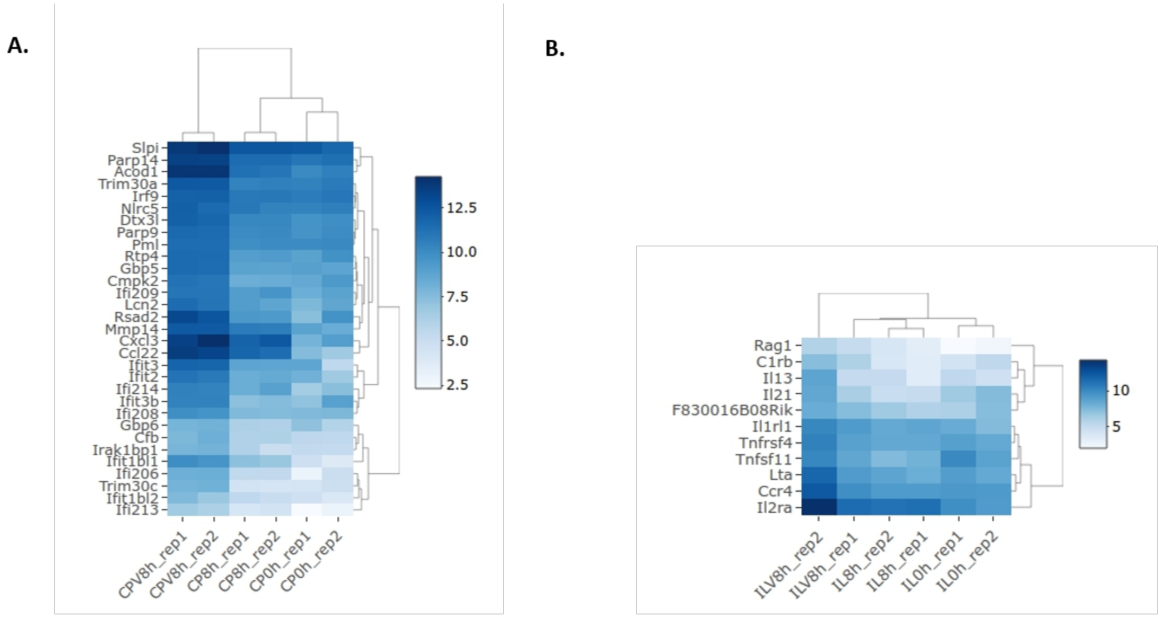

Lung cells collected from PBS vaccinated mice

Lung cells collected from 10 L-PaF/BECC/ME mice

Supplementary Figure 5. Heatmaps showing the pattern of gene upregulation following PcrV treatment in lung cells from PBS or 10 L-PaF BECC/ME vaccinated mice. CPV8h_rep1 = PcrV treated (for 8 hours) cells from control or PBS vaccinated mouse_sample #1, CPV8h_rep2 = PcrV treated (for 8 hours) cells from control or PBS vaccinated mouse_sample #2, CP8h_rep1 = Lung cells from control or PBS vaccinated mice with no treatment (for 8 hours)_sample #1, CP8h_rep2 = Lung cells from control or PBS vaccinated mice with no treatment (for 8 hours)_sample #2, CP0h_rep1 = Lung cells from control or PBS vaccinated mice with no treatment, no incubation (0 hour)_sample #1, CP0h_rep2 = Lung cells control or PBS vaccinated mice with no treatment, no incubation (o hour)_sample #2. Similarly, ILV8h_rep1 is same as CPV8h_rep1 except the cells were collected from immunized or 10 µg L-PaF BECC/ME mice and so on. Genes from control or PBS vaccinated mice are shown in **(A)**, genes from immunized or 10 µg L-PaF BECC/ME vaccinated mice are shown in **(B)**.

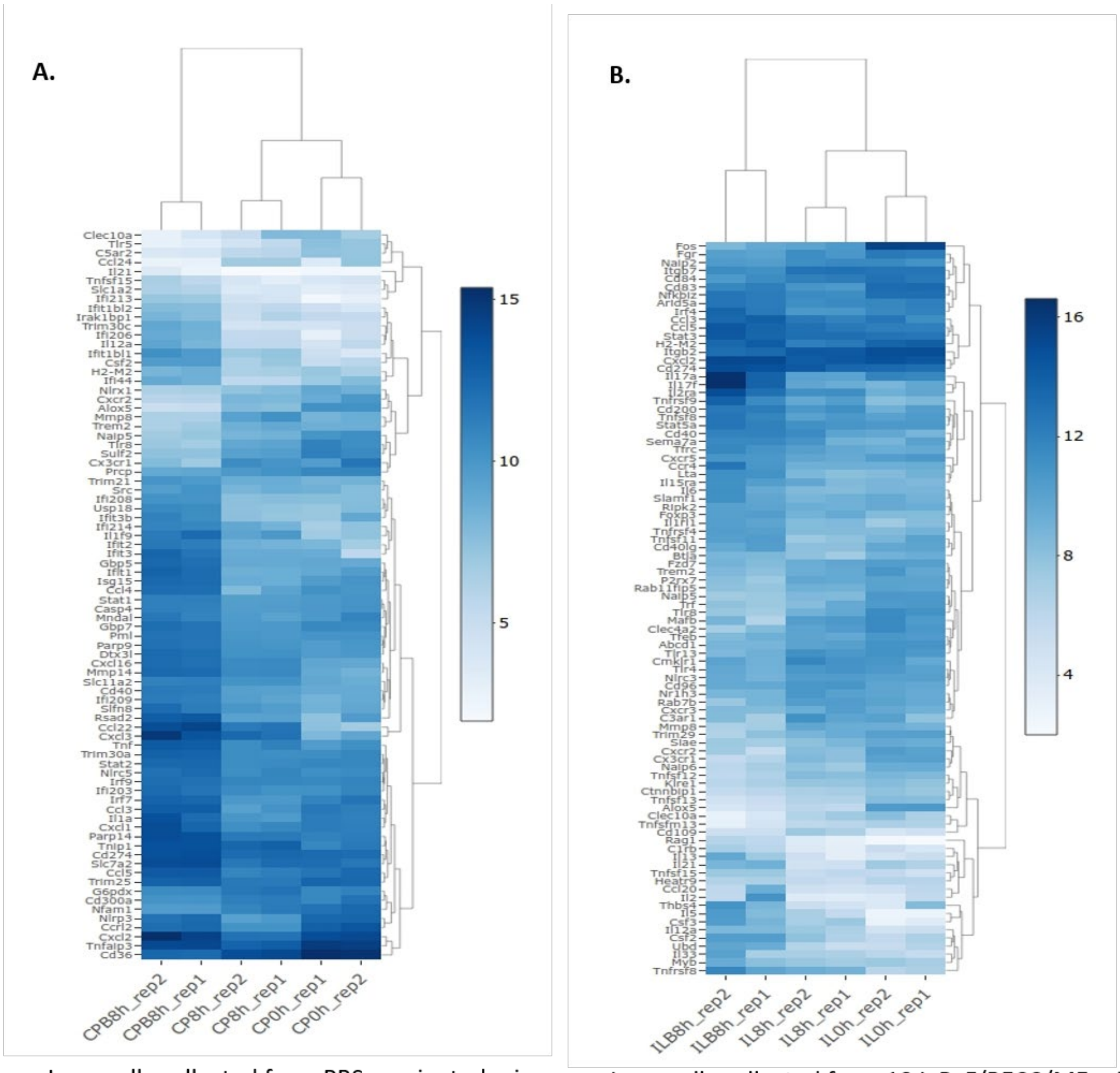

Lung cells collected from PBS vaccinated mice

Lung cells collected from 10 L-PaF/BECC/ME mice

Supplementary Figure 6. Heatmaps showing the pattern of gene upregulation following PopB treatment in lung cells from PBS or 10 L-PaFBECC/ME vaccinated mice. The groups are same as **Fig. S3**, except PopB was used here, instead of PcrV. Genes from PBS vaccinated mice are shown in **(A)**, genes from PBS vaccinated mice are shown in **(B)**.

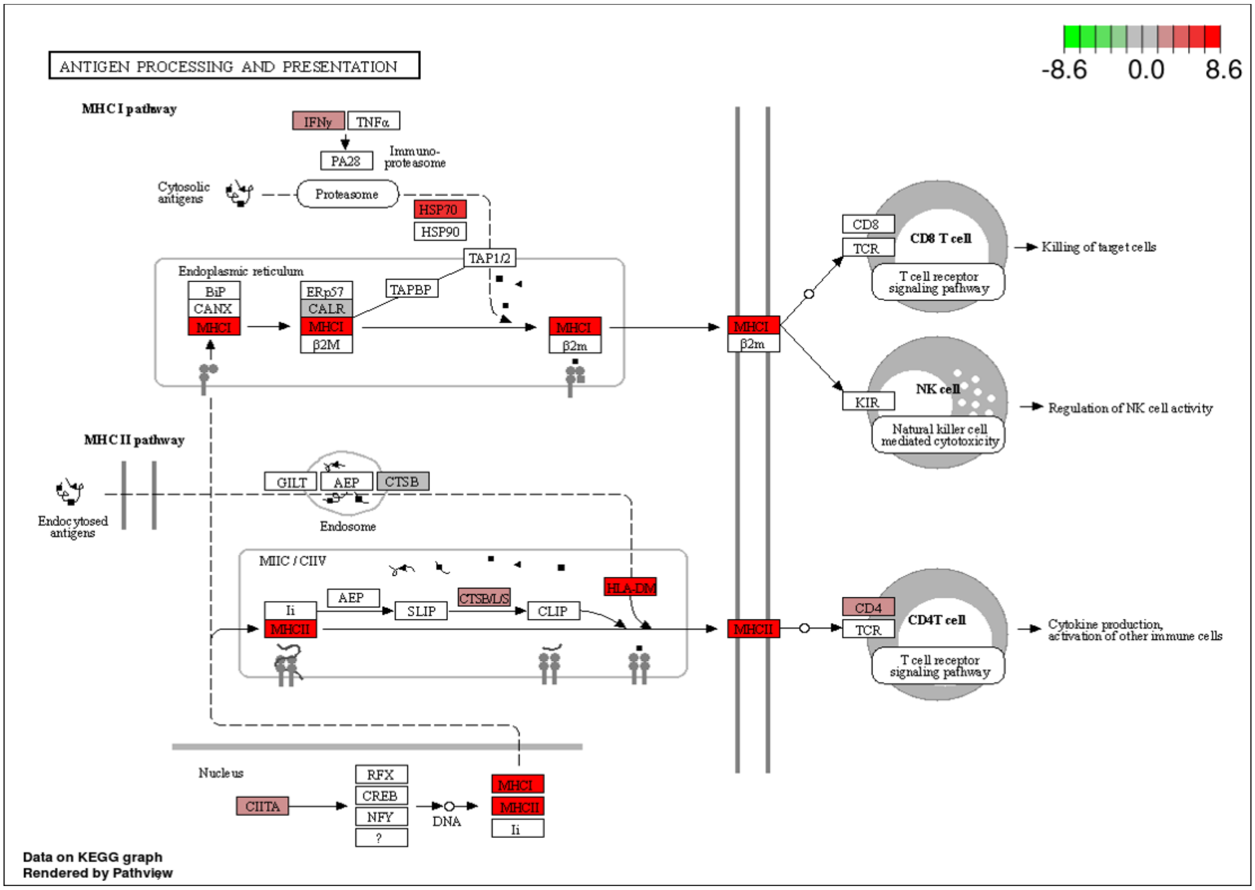

Supplementary Figure 7. Antigen processing and presentation. Upregulated genes from this pathway have been shown. A scale from -8.6 to +8.6 was used.

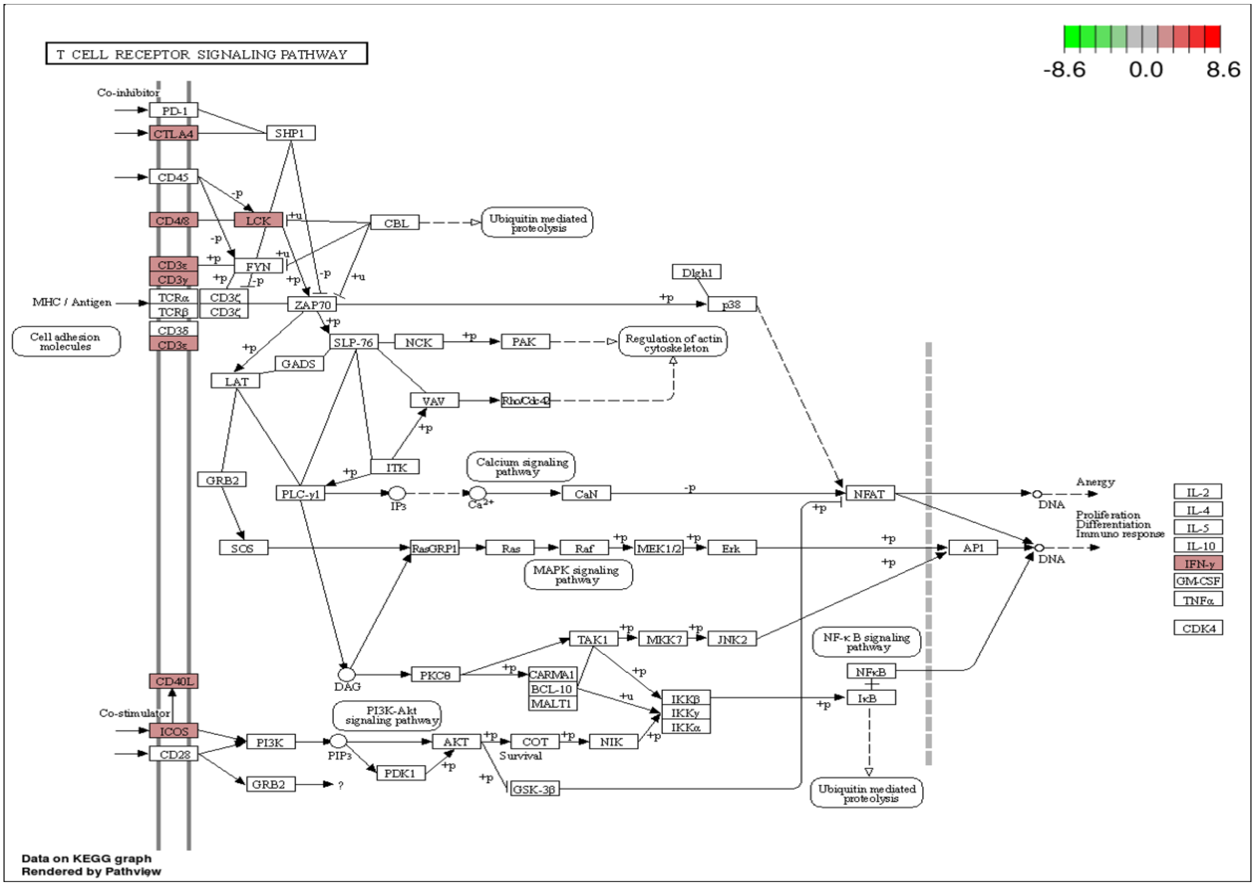

Supplementary Figure 8. TCR signaling. Upregulated genes from this pathway have been shown. A scale from -8.6 to +8.6 was used.

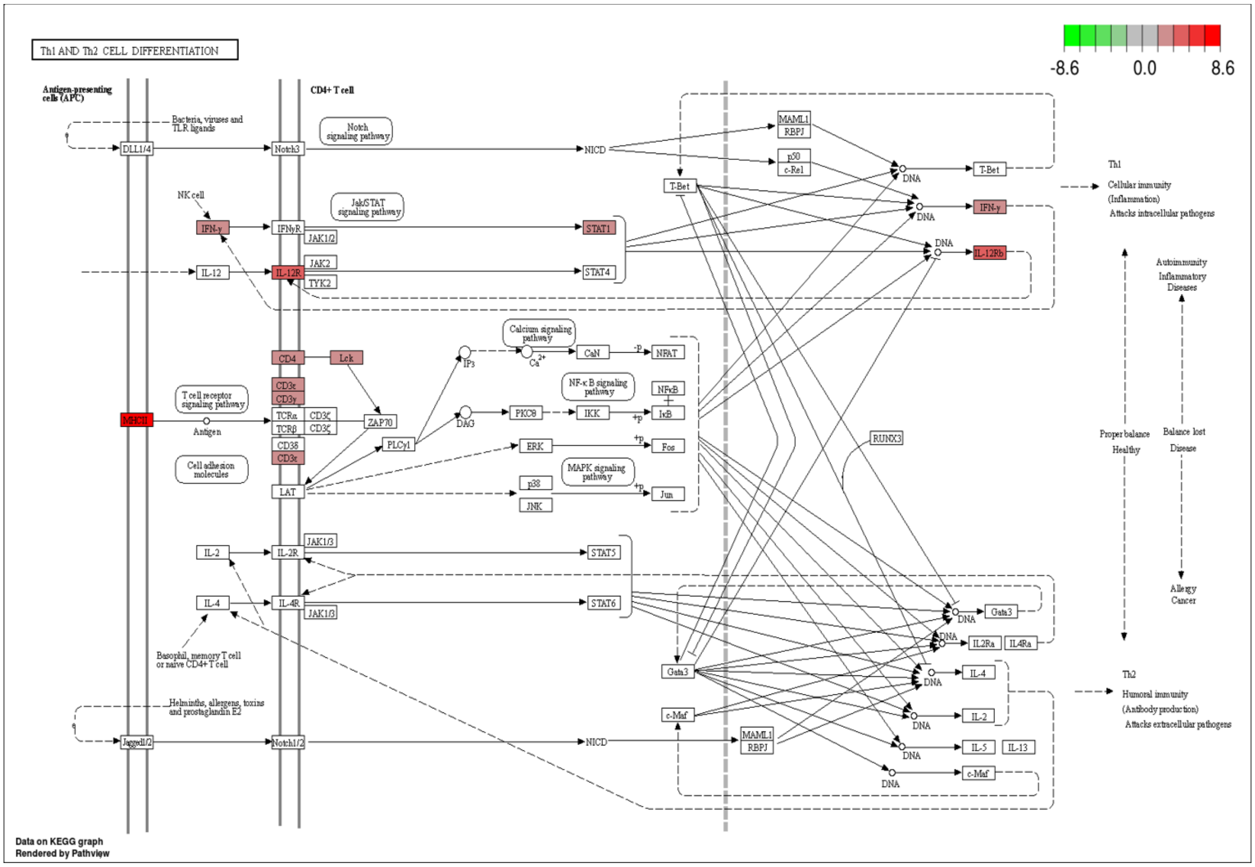

Supplementary Figure 9. Th1/Th2 differentiation. Upregulated genes from this pathway have been shown. A scale from -8.6 to +8.6 was used.

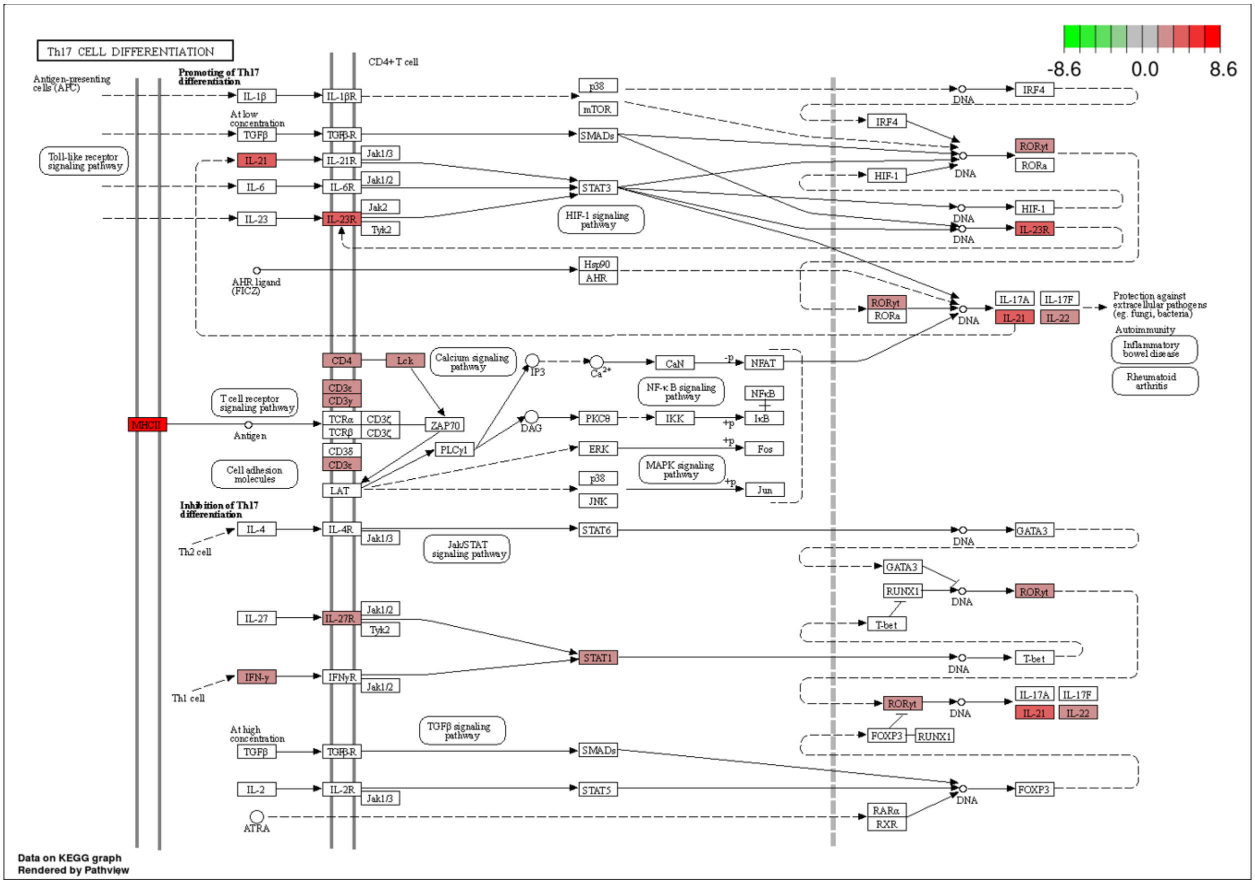

Supplementary Figure 10. Th17 signaling. Upregulated genes from this pathway have been shown. A scale from -8.6 to +8.6 was used.

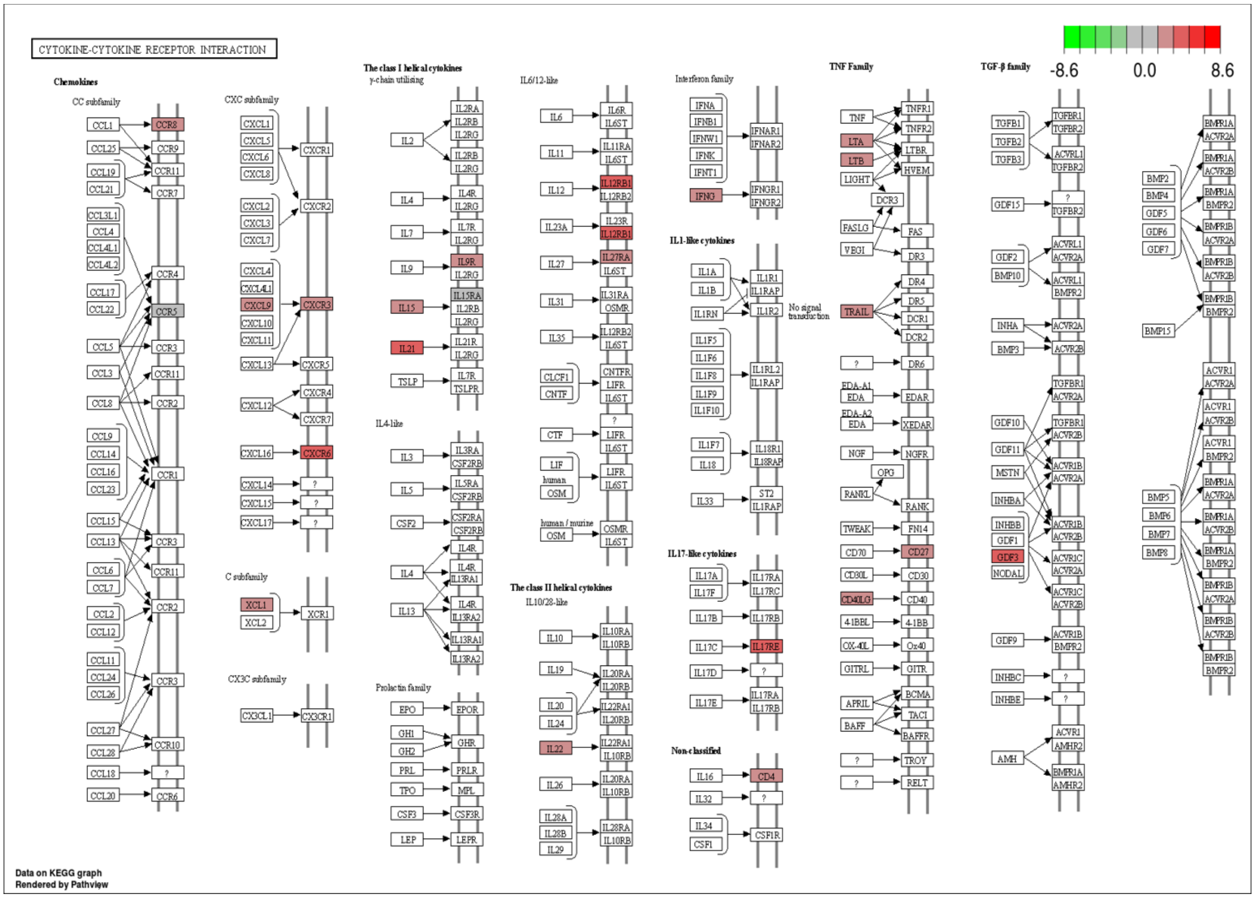

Supplementary Figure 11. Cytokine-cytokine receptor interaction. Upregulated genes from this pathway have been shown. A scale from -8.6 to +8.6 was used.

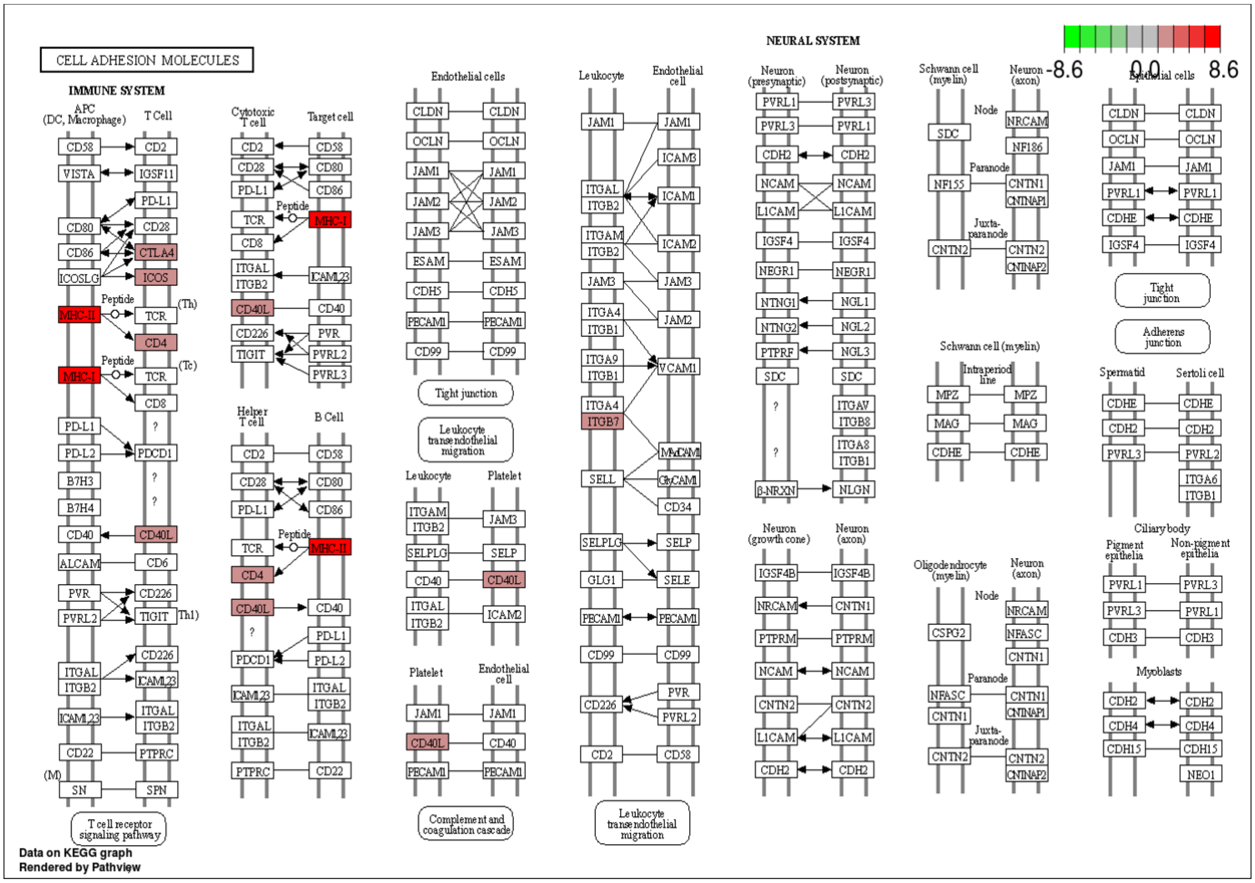

Supplementary Figure 12. Cell adhesion molecules. Upregulated genes from this pathway have been shown. A scale from -8.6 to +8.6 was used.

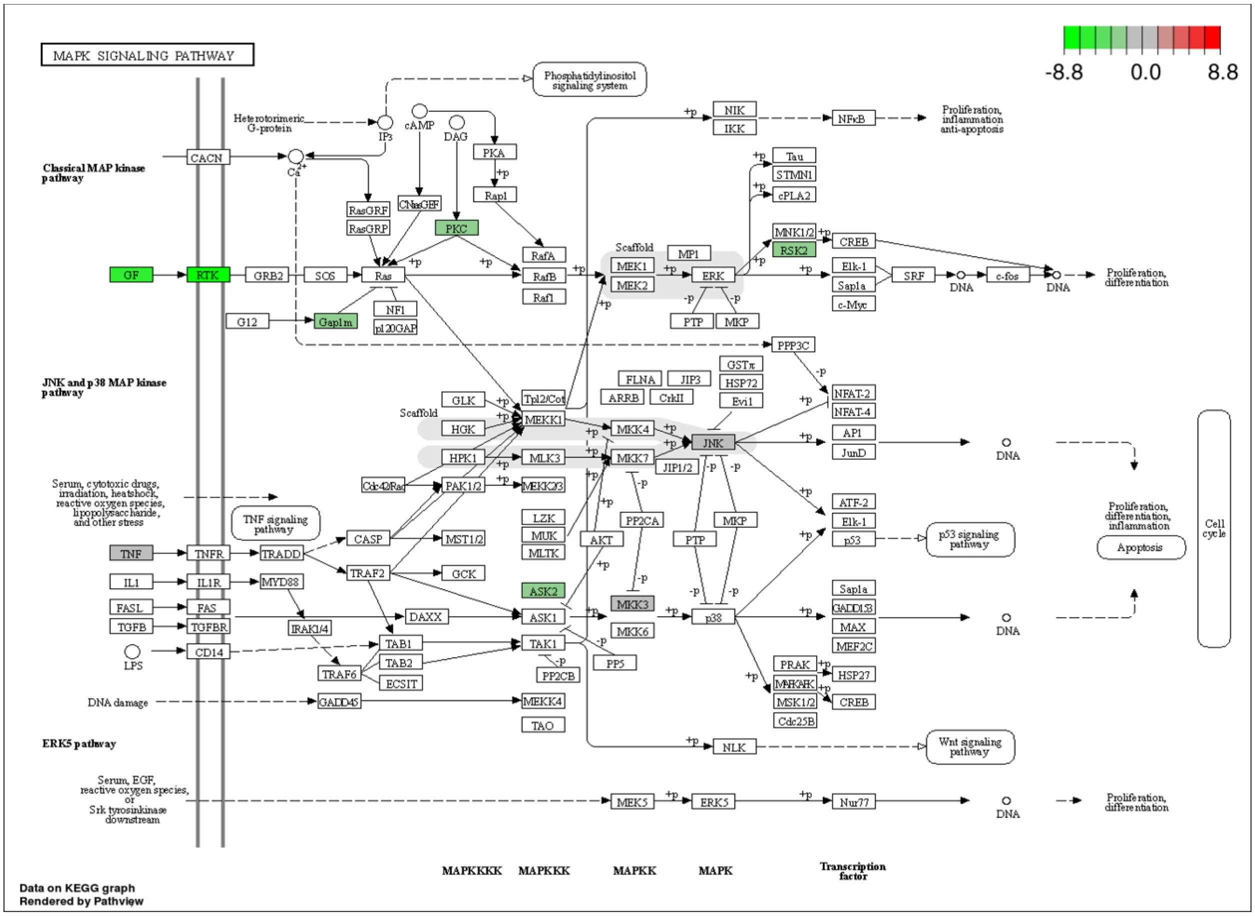

Supplementary Figure 13. MAPK signaling. Downregulated genes from this pathway have been shown. A scale from -8.8 to +8.8 was used.

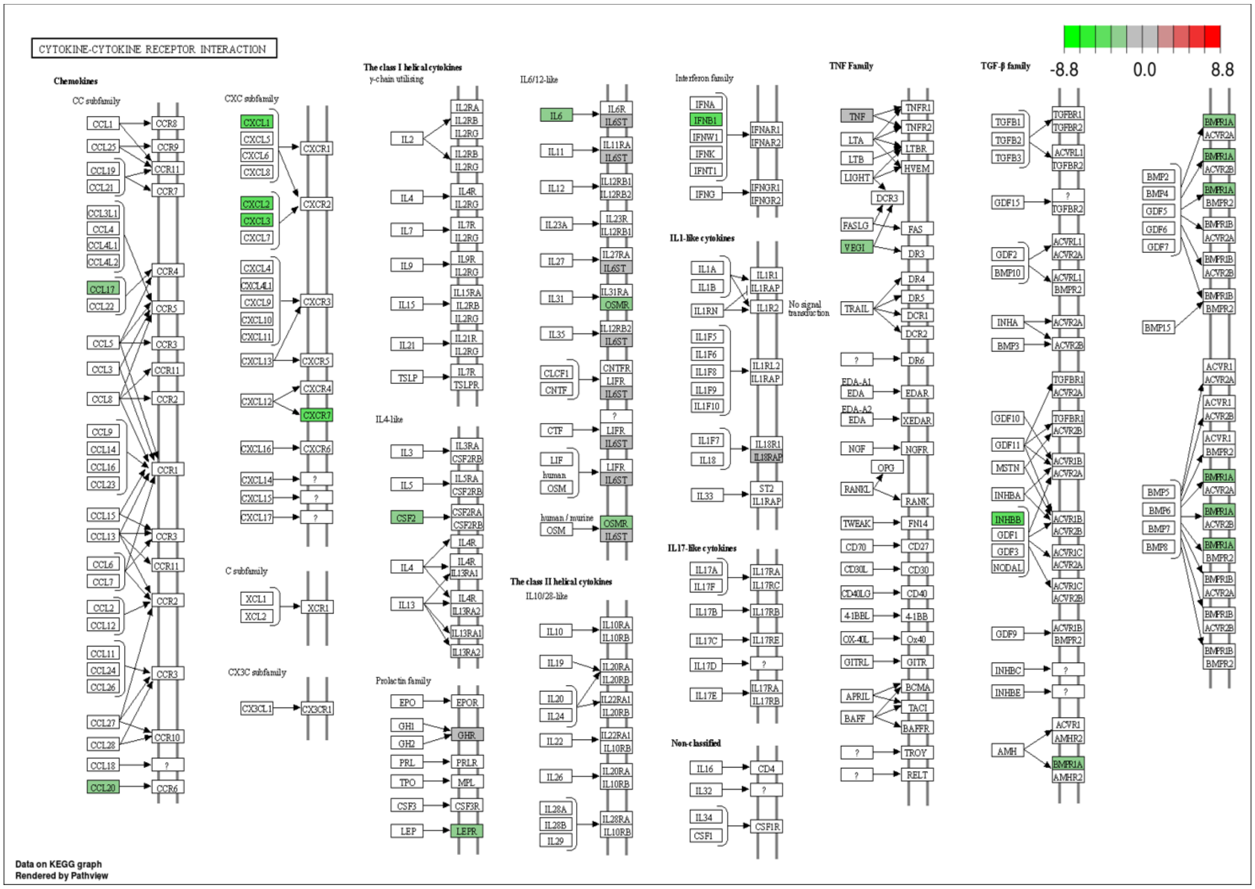

Supplementary Figure 14. Cytokine-cytokine receptor interaction. Downregulated genes from this pathway have been shown. A scale from -8.8 to +8.8 was used.

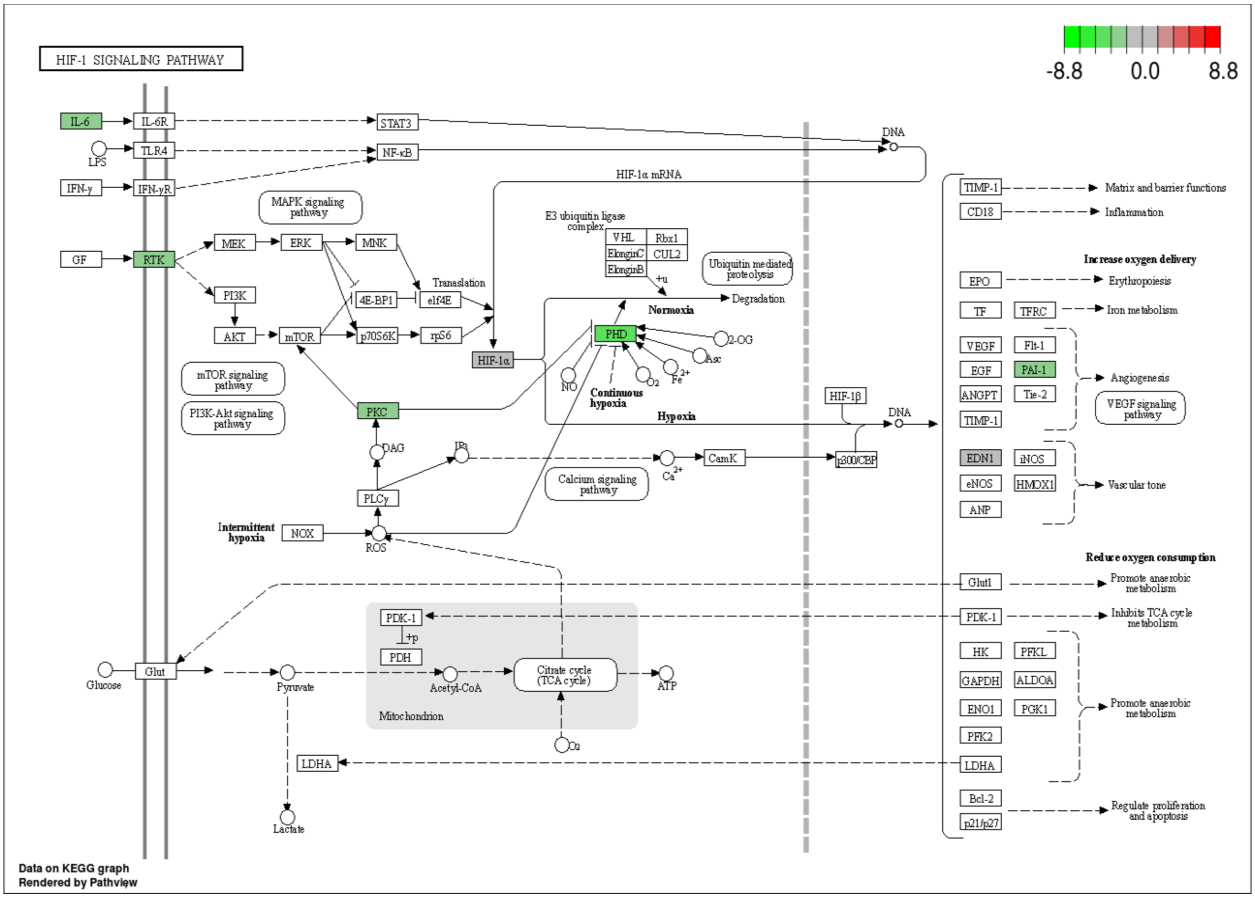

Supplementary Figure 15. HIF-1 signaling. Downregulated genes from this pathway have been shown. A scale from -8.8 to +8.8 was used.

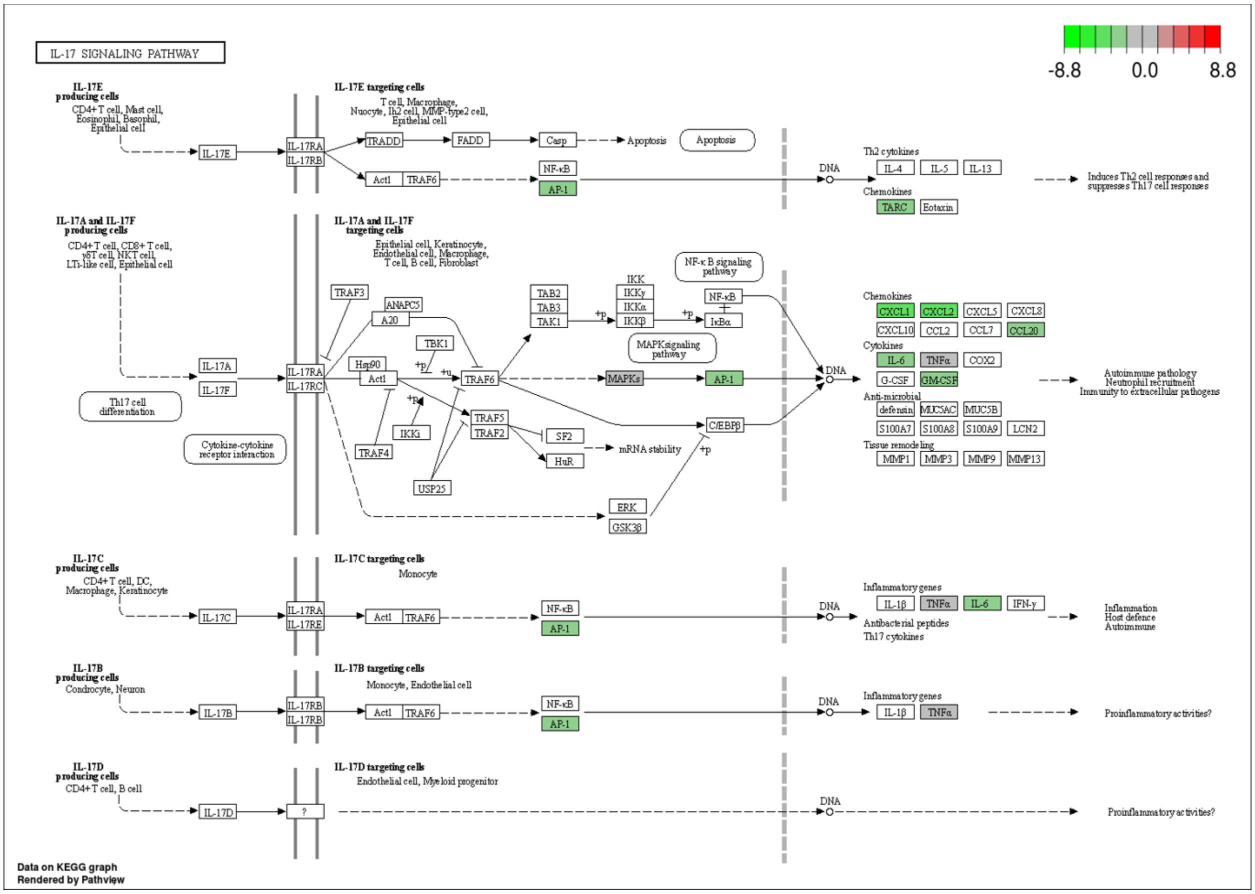

Supplementary Figure 16. IL-17 signaling. Downregulated genes from this pathway have been shown. A scale from -8.8 to +8.8 was used.

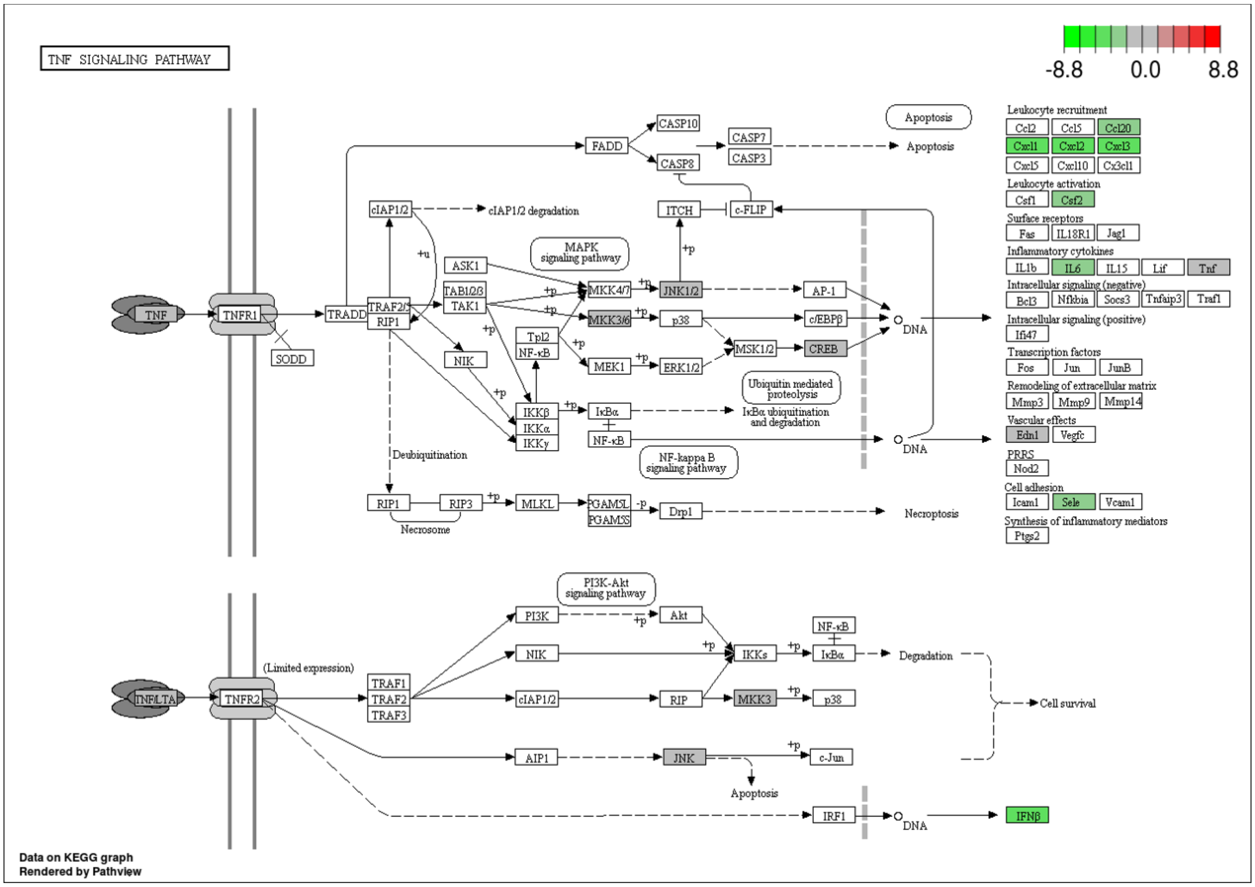

Supplementary Figure 17. TNF signaling. Downregulated genes from this pathway have been shown. A scale from -8.8 to +8.8 was used.

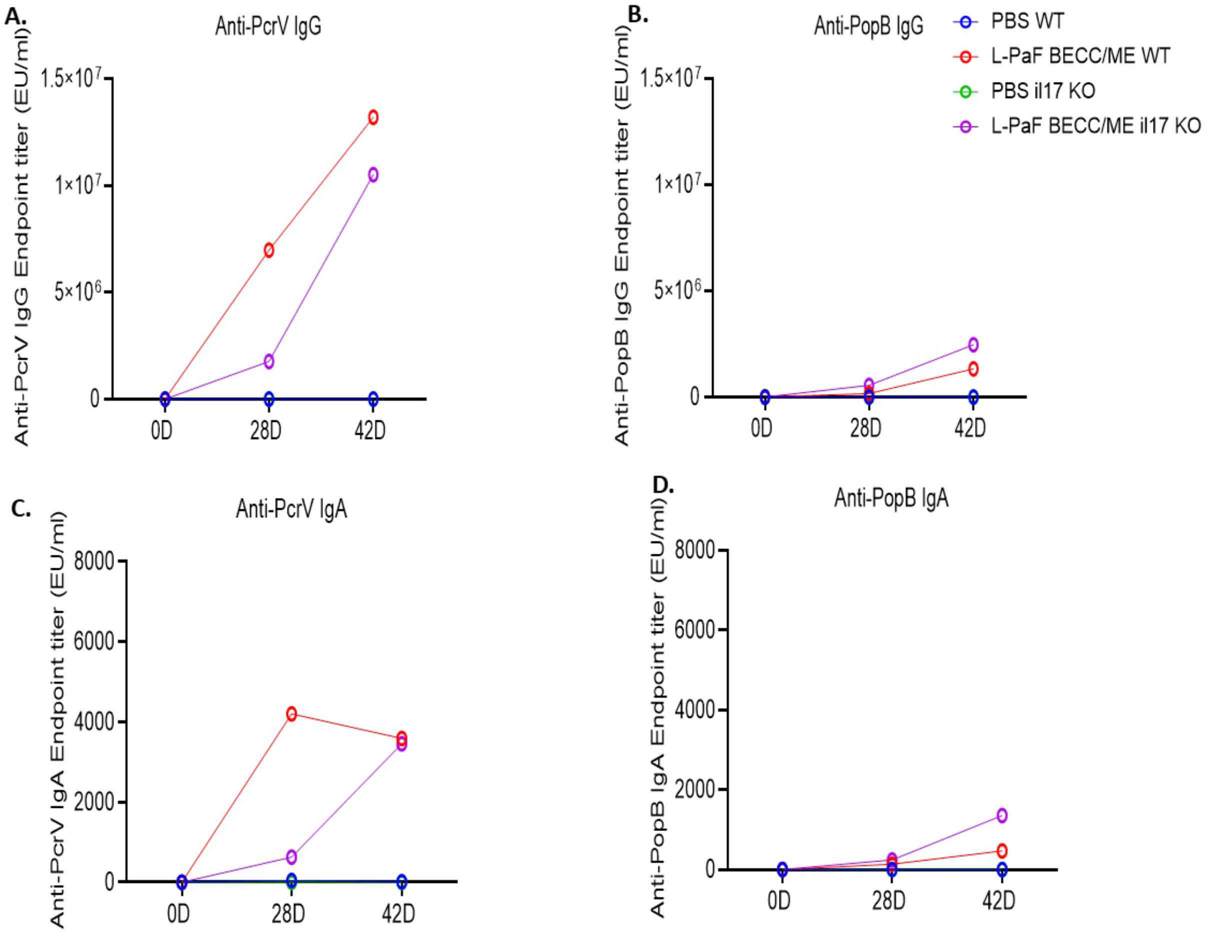

Supplementary Figure 18. Kinetics of serum IgG, IgA. B6.WT and IL-17 KO mice were vaccinated on days 0, 14 and 28 and their sera were assessed for anti-PcrV and/or anti-PopB immunoglobulins. Anti-PcrV IgG **(A)**, IgA **(C)**, and anti-PopB IgG **(B)**, IgA **(D)**, are shown. Titers represented as EU/ml. Each point denotes a titer value from a pooled serum.

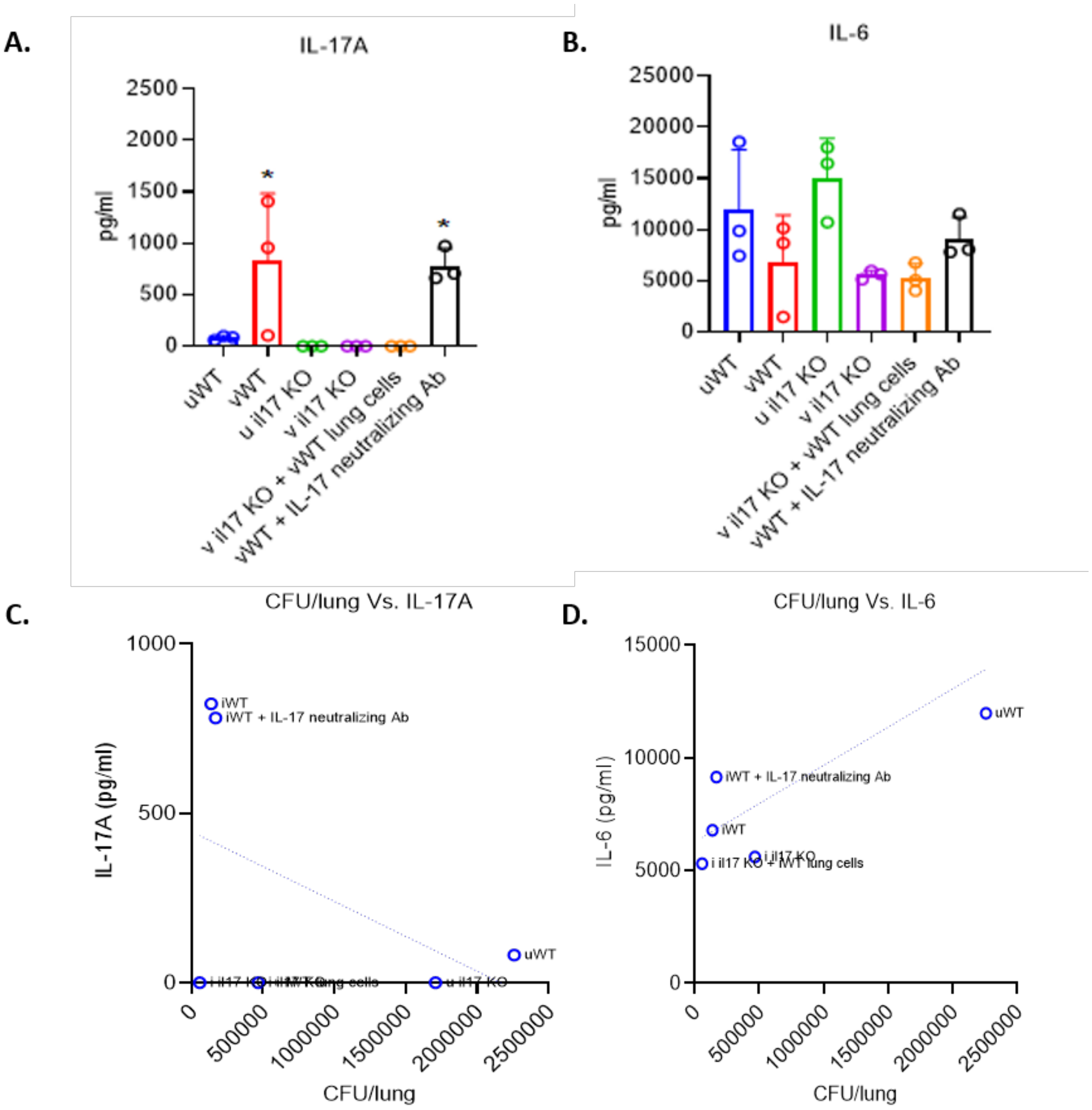

Supplementary Figure 19. Correlation between lung burden and lung cytokines. Lung cell suspensions obtained from infected mice at 16 HPI, were prepared as described in the text and left untreated for 48 hours at 37°C. Secretion of IL-17A **(A)** and IL-6 **(B)** were measured by MesoScale Discovery (MSD) as per the manufacturer's instructions. Each dot represents actual values and error bars shows SD (n = 3/group). The values were compared with PBS using a two-way ANOVA (Dunnett's test). *$p < 0.05$. Correlation analysis was done in terms of lung burden Vs. post-challenge IL-17A **(C)** and lung burden

Vs. post-challenge IL-6 **(D)**. Pearson's r coefficient and simple linear regression (95% confidence level) were calculated. r = -0.4806, 95% confidence interval = -0.9296 to 0.5426, R squared = 0.2309, p value (two-tailed) = 0.335 for **(C)** and r = 0.8222, 95% confidence interval = 0.03186 to 0.9799, R squared = 0.6760, p value (two-tailed) = 0.045 was observed.

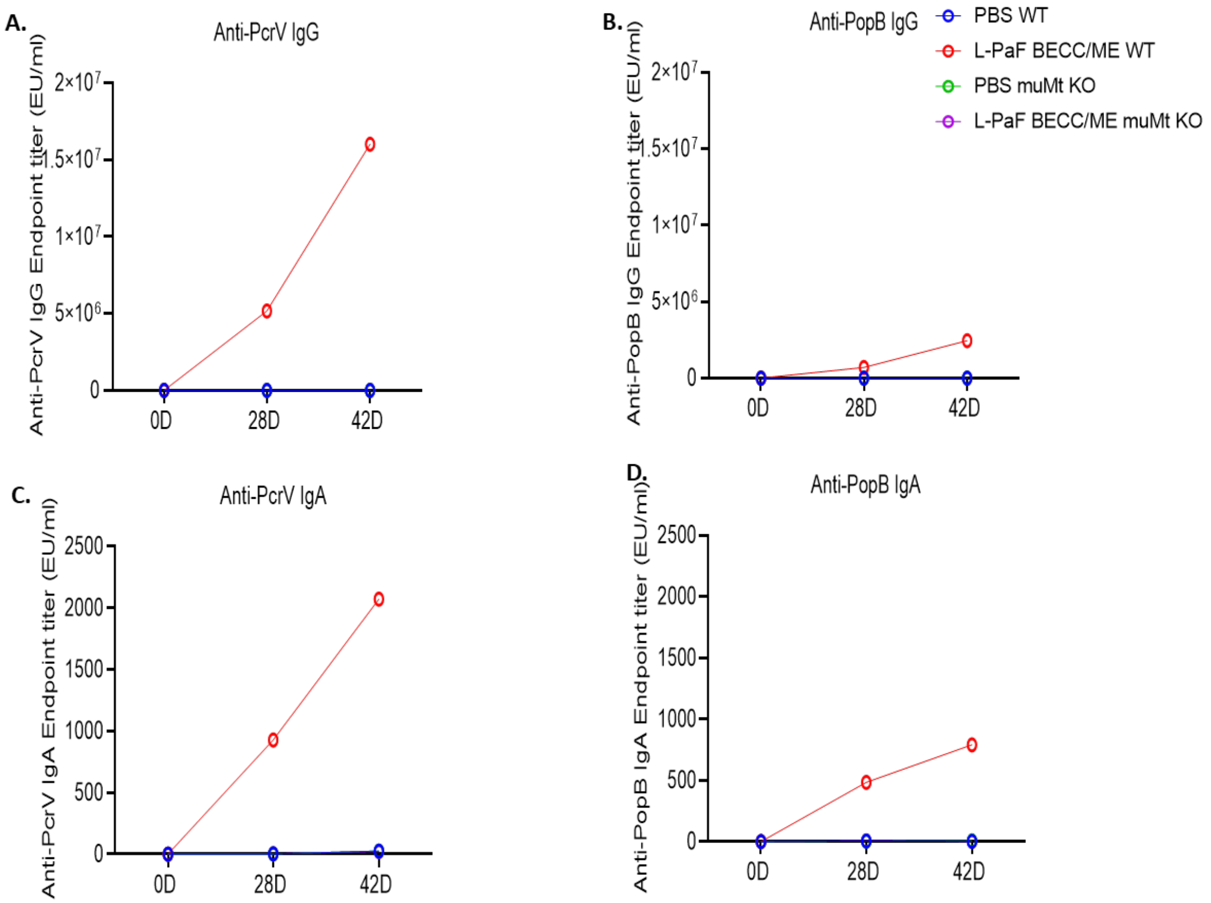

Supplementary Figure 20. Kinetics of serum IgG, IgA. B6.WT and muMt$^-$ KO mice were vaccinated on days 0, 14 and 28 and their sera were assessed for anti-PcrV and/or anti-PopB immunoglobulins. Anti-PcrV IgG **(A)**, IgA **(C)**, and anti-PopB IgG **(B)**, IgA **(D)**, are shown. Titers represented as EU/ml. Each point denotes a titer value from a pooled serum.

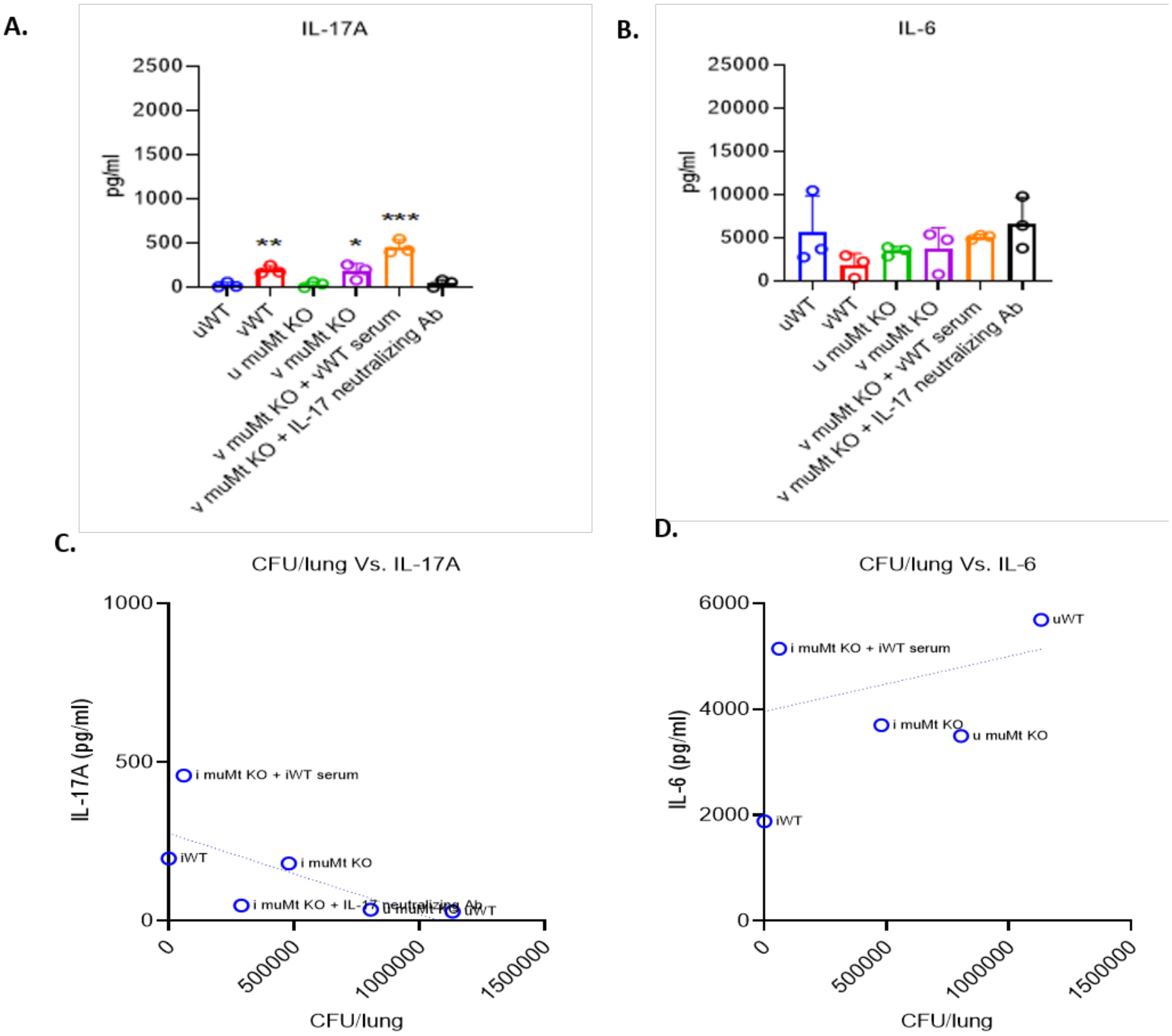

Supplementary Figure 21. Correlation between lung burden and lung cytokines. Lung cell suspensions obtained from infected mice at 16 HPI, were prepared as described in the text and left untreated for 48 hours at 37°C. Secretion of IL-17A **(A)** and IL-6 **(B)** were measured by MesoScale Discovery (MSD) as per the manufacturer's instructions. Each dot represents actual values and error bars shows SD (n = 3/group). The values were compared with PBS using a two-way ANOVA (Dunnett's test). *p < 0.05, **p < 0.01, ***p < 0.001. Correlation analysis was done in terms of lung burden Vs. post-challenge IL-17A **(C)** and lung burden Vs. post-challenge IL-6 **(D)**. Pearson's r coefficient and simple

linear regression (95% confidence level) were calculated. r = -0.6819, 95% confidence interval = -0.9614 to 0.2904, R squared = 0.4649, p value (two-tailed) = 0.136 for **(C)** and r = 0.2626, 95% confidence interval = -0.6976 to 0.8855, R squared = 0.06896, p value (two-tailed) = 0.615 was observed.

Supplementary Tables.

Supplementary Table 1.

	OPK vs. Anti-PcrV IgG	OPK vs. Anti-PopB IgG	OPK vs. Anti-PcrV IgA	OPK vs. Anti-PopB IgA
Pearson r				
r	0.8378	0.8357	0.8435	0.8653
95% confidence interval	0.3251 to 0.9699	0.3186 to 0.9695	0.3423 to 0.9710	0.4116 to 0.9753
R squared	0.7020	0.6983	0.7115	0.7487
P value				
P (two-tailed)	.009	.010	.008	.006
P value summary	**	**	**	**
Significant? (alpha = 0.05)	Yes	Yes	Yes	Yes

Supplementary Table 1. Correlation between OPK and anti-PcrV, anti-PopB IgG and IgAs (log10 scale). Correlation assays were done in GrapahPad and Pearson’s r was determined.

Supplementary Table 2.

	OPK vs. Anti-PcrV IgG1	OPK vs. Anti-PopB IgG1	OPK vs. Anti-PcrV IgG2a	OPK vs. Anti-PopB IgG2a	OPK vs. Anti-PcrV IgG3	OPK vs. Anti-PopB IgG3
Pearson r						
r	0.8276	0.8255	0.8423	0.8308	0.8216	0.7438
95% confidence interval	0.2949 to 0.9678	0.2888 to 0.9674	0.3387 to 0.9708	0.3042 to 0.9685	0.2778 to 0.9666	0.08218 to 0.9504
R squared	0.6849	0.6814	0.7095	0.6902	0.6751	0.5532
P value						
P (two-tailed)	.011	.012	.009	.011	.012	.034
P value summary	*	*	**	*	*	*
Significant? (alpha = 0.05)	Yes	Yes	Yes	Yes	Yes	Yes

Supplementary Table 2. Correlation between OPK and IgG subtypes (log10 scale) against PcrV and PopB. Correlation assays were done in GrapahPad and Pearson's r was determined.

Supplementary Table 3.

	CFU/lung vs. Pre-challenge PcrV (IL-17A)	CFU/lung vs. Pre-challenge PopB (IL-17A)	CFU/lung vs. Pre-challenge PcrV (IFN-γ)	CFU/lung vs. Pre-challenge PopB (IFN-γ)	CFU/lung vs. Pre-challenge PcrV (TNF-α)	CFU/lung vs. Pre-challenge PopB (TNF-α)	CFU/lung vs. Pre-challenge PcrV (IL-6)	CFU/lung vs. Pre-challenge PopB (IL-6)
Pearson r								
r	-0.9131	-0.9146	-0.8204	-0.8950	-0.7333	-0.5755	-0.6730	-0.6741
95% confidence interval	-0.9844 to -0.5844	-0.9847 to -0.5906	-0.9664 to -0.2742	-0.9810 to -0.5153	-0.9481 to -0.05919	-0.9108 to 0.2173	-0.9345 to 0.06031	-0.9348 to 0.05819
R squared	0.8337	0.8366	0.6730	0.8010	0.5377	0.3312	0.4529	0.4544
P value								
P (two-tailed)	.002	.001	.013	.003	.038	.136	.067	.067
P value summary	**	**	*	**	*	ns	ns	ns
Significant? (alpha = 0.05)	Yes	Yes	Yes	Yes	Yes	No	No	No

Supplementary Table 3. Correlation between lung burden (CFU/lung) and pre-challenge cytokines in MSD assay. Correlation assays were done in GrapahPad and Pearson's r was determined. Correlation assays were done in GrapahPad and Pearson's r was determined.

Supplementary Table 4. A.

	CFU/lung vs. post-challenge lung IL-17A	CFU/lung vs. post-challenge lung IFN-γ	CFU/lung vs. post-challenge TNF-α	CFU/lung vs. post-challenge IL-6
Pearson r				
r	0.9640	0.8513	0.9539	0.8175
95% confidence interval	0.8084 to 0.9937	0.3664 to 0.9726	0.7602 to 0.9919	0.2660 to 0.9658
R squared	0.9292	0.7247	0.9099	0.6682
P value				
P (two-tailed)	<.001	.007	<.001	.013
P value summary	***	**	***	*
Significant? (alpha = 0.05)	Yes	Yes	Yes	Yes

Supplementary Table 4. B.

IL-17A:

Pre IL-17A un st.	Post IL-17A un st.	Fold-change
6.799740023	1099.566817	161.7071849
12.4157399	1407.798205	113.3881844
1142.242666	2066.608956	1.809255615
656.9694475	1372.598294	2.089287864
807.0611429	1455.480914	1.803433268

121.2592195	1552.06946	12.79959963
339.6481756	2108.678407	6.208419648
1641.370134	3450.190909	2.102018818

IFN-γ:

Pre IFN-γ un st.	Post IFN-γ un st.	Fold-change
2.295041853	1960.908776	854.4109006
60.96101878	2156.966591	35.3827189
1680.190127	1858.129402	1.10590425
192.9786844	1764.409612	9.1430285
299.2313919	1257.441553	4.202238091
179.324218	2187.417891	12.19811756
1069.297221	1620.292623	1.515287416
1433.393006	2394.030756	1.670184483

TNF-α:

Pre TNF-α un st.	Post TNF-α un st.	Fold-change
266.99884	5086.699431	19.0513915
231.6189881	4003.352311	17.28421466
1269.543166	2147.483232	1.691540146
404.9621679	2562.495077	6.327739429
507.2675381	1748.715759	3.447324395
967.7809215	2580.956627	2.666881077
1302.613599	1743.583389	1.338526936
2014.159615	1771.265888	0.879406912

IL-6:

Pre IL-6 un st.	Post IL-6 un st.	Fold-change
673.2776262	11145.98277	16.55480939
526.223799	7556.153817	14.35920198
5959.122424	6367.876902	1.068593066
645.1086716	9766.800395	15.1397754

744.1124779	5237.644317	7.03878039
1470.297353	6848.788818	4.658097767
3962.071722	11735.63869	2.961995519
6208.595007	18314.05169	2.949790037

The numbers in pre- and post-columns are in pg/ml.

Abbr: un st. = unstimulated.

Supplementary Table 4. A. Correlation between lung burden (CFU/lung) and post-challenge cytokines in MSD assay. Correlation assays were done in GrapahPad and Pearson’s r was determined. **B.** Exact fold-change values of different post-challenge cytokines.

Supplementary Table 5.

	CFU/lung vs. Anti-PcrV IgG	CFU/lung vs. Anti-PopB IgG	CFU/lung vs. Anti-PcrV IgA	CFU/lung vs. Anti-PopB IgA
Pearson r				
r	-0.9406	-0.9399	-0.9349	-0.9353
95% confidence interval	-0.9895 to -0.6999	-0.9893 to -0.6968	-0.9884 to -0.6747	-0.9885 to -0.6767
R squared	0.8848	0.8835	0.8740	0.8749
P value				
P (two-tailed)	<.001	<.001	<.001	<.001
P value summary	***	***	***	***
Significant? (alpha = 0.05)	Yes	Yes	Yes	Yes

Supplementary Table 5. Correlation between lung burden and anti-PcrV, anti-PopB serum IgG, IgA. Correlation analyses were done using GraphPad and Pearson's r values were determined.

Supplementary Table 6.

	CFU/lung vs. Anti-PcrV IgG1	CFU/lung vs. Anti-PopB IgG1	CFU/lung vs. Anti-PcrV IgG2a	CFU/lung vs. Anti-PopB IgG2a	CFU/lung vs. Anti-PcrV IgG3	CFU/lung vs. Anti-PopB IgG3
Pearson r						
r	-0.9047	-0.9316	-0.9319	-0.9345	-0.9460	-0.8797
95% confidence interval	-0.9828 to -0.5517	-0.9878 to -0.6605	-0.9879 to -0.6618	-0.9883 to -0.6731	-0.9904 to -0.7239	-0.9781 to -0.4606
R squared	0.8184	0.8678	0.8684	0.8733	0.8949	0.7739
P value						
P (two-tailed)	.002	<.001	<.001	<.001	<.001	.004
P value summary	**	***	***	***	***	**
Significant? (alpha = 0.05)	Yes	Yes	Yes	Yes	Yes	Yes

Supplementary Table 6. Correlation between lung burden and different subtypes of IgG against PcrV and PopB. Correlation analyses were done using GraphPad and Pearson's r values were determined.

Supplementary Table 7.

	IL-17A vs. Anti-PcrV IgG1	IL-17A vs. Anti-PopB IgG1	IL-17A vs. Anti-PcrV IgG2a	IL-17A vs. Anti-PopB IgG2a	IL-17A vs. Anti-PcrV IgG3	IL-17A vs. Anti-PopB IgG3
Pearson r						
r	0.9266	0.9504	0.9362	0.9341	0.9388	0.8974
95% confidence interval	0.6396 to 0.9869	0.7438 to 0.9912	0.6806 to 0.9887	0.6713 to 0.9883	0.6917 to 0.9891	0.5243 to 0.9814
R squared	0.8586	0.9032	0.8766	0.8725	0.8813	0.8053
P value						
P (two-tailed)	<.001	<.001	<.001	<.001	<.001	.002
P value summary	***	***	***	***	***	**
Significant? (alpha = 0.05)	Yes	Yes	Yes	Yes	Yes	Yes

Supplementary Table 7. Correlation between post-challenge IL-17A from untreated lung and different subtypes of IgG against PcrV and PopB. Correlation analyses have been done using GraphPad and Pearson's r values were determined.
