# Supplementary material for: A protein subunit vaccine elicits a balanced immune response that protects against Pseudomonas pulmonary infection
Source: NPJ Vaccines. 2023 Mar 14;8:37. doi: 10.1038/s41541-023-00618-w (PMC10012293; doi:10.1038/s41541-023-00618-w)
Supplement: Supplementary file 2 — REPORTING SUMMARY [file 41541_2023_618_MOESM2_ESM.pdf]

nature portfolio

Corresponding author(s): *Wendy Picking*

Last updated by author(s): *Jan 20, 2023*

Reporting Summary

Nature Portfolio wishes to improve the reproducibility of the work that we publish. This form provides structure for consistency and transparency in reporting. For further information on Nature Portfolio policies, see our Editorial Policies and the Editorial Policy Checklist.

Statistics

For all statistical analyses, confirm that the following items are present in the figure legend, table legend, main text, or Methods section.

n/a	Confirmed	
☐	☒	The exact sample size (*n*) for each experimental group/condition, given as a discrete number and unit of measurement
☐	☒	A statement on whether measurements were taken from distinct samples or whether the same sample was measured repeatedly
☐	☒	The statistical test(s) used AND whether they are one- or two-sided<br>*Only common tests should be described solely by name; describe more complex techniques in the Methods section.*
☐	☒	A description of all covariates tested
☐	☒	A description of any assumptions or corrections, such as tests of normality and adjustment for multiple comparisons
☐	☒	A full description of the statistical parameters including central tendency (e.g. means) or other basic estimates (e.g. regression coefficient) AND variation (e.g. standard deviation) or associated estimates of uncertainty (e.g. confidence intervals)
☐	☒	For null hypothesis testing, the test statistic (e.g. *F*, *t*, *r*) with confidence intervals, effect sizes, degrees of freedom and *P* value noted<br>*Give P values as exact values whenever suitable.*
☒	☐	For Bayesian analysis, information on the choice of priors and Markov chain Monte Carlo settings
☒	☐	For hierarchical and complex designs, identification of the appropriate level for tests and full reporting of outcomes
☐	☒	Estimates of effect sizes (e.g. Cohen's *d*, Pearson's *r*), indicating how they were calculated

Our web collection on statistics for biologists contains articles on many of the points above.

Software and code

Policy information about availability of computer code

Data collection	*mRNA-seq read count data was provided by Novogene.*
Data analysis	*The mRNA seq. data were further processed using the iDEP web server (PMID: 30567491) for differential expression and pathway analysis. Initially, only the genes with at least 0.5 counts per million (CPM) reads in at least one sample were considered. Read counts were then transformed as log2(CPM) using the EdgeR method. The differentially expressed genes (DEGs) were identified using DESeq2 with an FDR cutoff 0.05 and a minimum fold change of 2. Finally, the enriched pathways in DEGs for the selected comparisons were identified through GO Biological Process analysis. Up-regulated pathways which most likely play a critical role in immune modulation were manually selected for each comparison. The fold change of individual genes associated with selected pathways were visualized as a heatmap using R. Gene Set Enrichment Analysis of Gene Ontology and KEGG pathway analysis was performed using R clusterProfiler (version 3.0.4).*<br><br>*GraphPad Prism 8.1.2 was used to prepare data and perform statistical analyses. PBS vaccinated groups were compared with the other vaccinated groups using Dunnett's multiple comparison test. A p value of < 0.05 was considered significant (*p < 0.05, **p < 0.01, ***p < 0.001). Pearson's r values and R squared values are mentioned as deemed appropriate.*

For manuscripts utilizing custom algorithms or software that are central to the research but not yet described in published literature, software must be made available to editors and reviewers. We strongly encourage code deposition in a community repository (e.g. GitHub). See the Nature Portfolio guidelines for submitting code & software for further information.

Data

Policy information about availability of data

All manuscripts must include a data availability statement. This statement should provide the following information, where applicable:

- Accession codes, unique identifiers, or web links for publicly available datasets
- A description of any restrictions on data availability
- For clinical datasets or third party data, please ensure that the statement adheres to our policy

Data will be available upon request.

Human research participants

Policy information about studies involving human research participants and Sex and Gender in Research.

Reporting on sex and gender	*NA.*
Population characteristics	*NA.*
Recruitment	*NA.*
Ethics oversight	*NA.*

Note that full information on the approval of the study protocol must also be provided in the manuscript.

Field-specific reporting

Please select the one below that is the best fit for your research. If you are not sure, read the appropriate sections before making your selection.

☒ Life sciences ☐ Behavioural & social sciences ☐ Ecological, evolutionary & environmental sciences

For a reference copy of the document with all sections, see nature.com/documents/nr-reporting-summary-flat.pdf

Life sciences study design

All studies must disclose on these points even when the disclosure is negative.

Sample size	*We carried out power calculations.*
Data exclusions	*No data were excluded.*
Replication	*Experiments were done with biological and technical triplicates.*
Randomization	*Naive mice collected from CRL were used in this study. They were randomly divided into either PBS-vaccinated or other vaccinated groups.*
Blinding	*NA.*

Behavioural & social sciences study design

All studies must disclose on these points even when the disclosure is negative.

Study description	*NA.*
Research sample	*NA.*
Sampling strategy	*NA.*
Data collection	*NA.*
Timing	*NA.*

Data exclusions	NA.
Non-participation	NA.
Randomization	NA.

Ecological, evolutionary & environmental sciences study design

All studies must disclose on these points even when the disclosure is negative.

Study description	NA.
Research sample	NA.
Sampling strategy	NA.
Data collection	NA.
Timing and spatial scale	NA.
Data exclusions	NA.
Reproducibility	NA.
Randomization	NA.
Blinding	NA.

Did the study involve field work? ☐ Yes ☒ No

Field work, collection and transport

Field conditions	NA.
Location	NA.
Access & import/export	NA.
Disturbance	NA.

Reporting for specific materials, systems and methods

We require information from authors about some types of materials, experimental systems and methods used in many studies. Here, indicate whether each material, system or method listed is relevant to your study. If you are not sure if a list item applies to your research, read the appropriate section before selecting a response.

Materials & experimental systems

n/a	Involved in the study	
☐	☒	Antibodies
☐	☒	Eukaryotic cell lines
☒	☐	Palaeontology and archaeology
☐	☒	Animals and other organisms
☒	☐	Clinical data
☒	☐	Dual use research of concern

Methods

n/a	Involved in the study	
☒	☐	ChIP-seq
☒	☐	Flow cytometry
☒	☐	MRI-based neuroimaging

Antibodies

Antibodies used	IgA: Cat. #: OB1040-05 (Southern biotech), IgG: Cat. #: 5450-0011(474-1806) (Sera Care). All secondary antibodies were horseradish

Antibodies used	*peroxidase (HRP)-labeled goat anti-mouse IgG/IgA, that were human-adsorbed.<br><br>IL-17A Rat anti-Mouse, NA/LE, Unlabeled, Clone: TC11-18H10, Cat. #: BDB560268.*
Validation	*All the antibodies are readily used by us and other researchers in the field. We used the same antibodies for ELISA in all of our previous studies and publications.*

Eukaryotic cell lines

Policy information about cell lines and Sex and Gender in Research

Cell line source(s)	*Primary mouse lung cells were isolated and single cell suspensions were prepared for ELISpot, MSD studies. J774.1 macrophage cell line was bought from ATCC.*
Authentication	*We have been extracting and using mouse lung cells for all our studies and have published several papers before. The J774.1 cell lines were supplied by ATCC and thus authenticated by the company.*
Mycoplasma contamination	*The cell lines were not tested for mycoplasma contamination. No unusual growths were observed during the procedures. The mouse primary cells were kept not more than 48 hours, whereas, the J774.1 cell line was passaged once before the experiment.*
Commonly misidentified lines (See ICLAC register)	*NA.*

Palaeontology and Archaeology

Specimen provenance	*NA.*
Specimen deposition	*NA.*
Dating methods	*NA.*

☐ Tick this box to confirm that the raw and calibrated dates are available in the paper or in Supplementary Information.

Ethics oversight	*NA.*

Note that full information on the approval of the study protocol must also be provided in the manuscript.

Animals and other research organisms

Policy information about studies involving animals; ARRIVE guidelines recommended for reporting animal research, and Sex and Gender in Research

Laboratory animals	*Six- to eight-week-old CD-1, C57BL/6 (B6), B6.Cg-Il17a/Il17ftm1.1Impr Thy1a/J (il17-/- or IL-17 KO) and B6.129S2-Ighmtm1Cgn/J (muMt- KO) mice were purchased from Charles River Laboratories (Wilmington, MA) or The Jackson Laboratory (ME, USA).*
Wild animals	*NA.*
Reporting on sex	*All female mice were used.*
Field-collected samples	*NA.*
Ethics oversight	*Animal works were carried out according to the University of Kansas (KU Lawrence) IACUC animal use statement (AUS 222-03, valid until March 9th, 2025). The Institution's Animal Welfare Assurance number is D16-00220 (A3339-01).*

Note that full information on the approval of the study protocol must also be provided in the manuscript.

Clinical data

Policy information about clinical studies

All manuscripts should comply with the ICMJE guidelines for publication of clinical research and a completed CONSORT checklist must be included with all submissions.

Clinical trial registration	*NA.*
Study protocol	*NA.*
Data collection	*NA.*
Outcomes	*NA.*

Dual use research of concern

Policy information about dual use research of concern

Hazards

Could the accidental, deliberate or reckless misuse of agents or technologies generated in the work, or the application of information presented in the manuscript, pose a threat to:

No	Yes	
☒	☐	Public health
☒	☐	National security
☒	☐	Crops and/or livestock
☒	☐	Ecosystems
☒	☐	Any other significant area

Experiments of concern

Does the work involve any of these experiments of concern:

No	Yes	
☒	☐	Demonstrate how to render a vaccine ineffective
☒	☐	Confer resistance to therapeutically useful antibiotics or antiviral agents
☒	☐	Enhance the virulence of a pathogen or render a nonpathogen virulent
☒	☐	Increase transmissibility of a pathogen
☒	☐	Alter the host range of a pathogen
☒	☐	Enable evasion of diagnostic/detection modalities
☒	☐	Enable the weaponization of a biological agent or toxin
☒	☐	Any other potentially harmful combination of experiments and agents

ChIP-seq

Data deposition

☐ Confirm that both raw and final processed data have been deposited in a public database such as GEO.

☐ Confirm that you have deposited or provided access to graph files (e.g. BED files) for the called peaks.

Data access links<br>*May remain private before publication.*	*NA.*
Files in database submission	*NA.*
Genome browser session<br>(e.g. UCSC)	*NA.*

Methodology

Replicates	*NA.*
Sequencing depth	*NA.*
Antibodies	*NA.*
Peak calling parameters	*NA.*
Data quality	*NA.*
Software	*NA .*

Flow Cytometry

Plots

Confirm that:

☐ The axis labels state the marker and fluorochrome used (e.g. CD4-FITC).

☐ The axis scales are clearly visible. Include numbers along axes only for bottom left plot of group (a 'group' is an analysis of identical markers).

☐ All plots are contour plots with outliers or pseudocolor plots.

☐ A numerical value for number of cells or percentage (with statistics) is provided.

Methodology

Sample preparation	NA.
Instrument	NA.
Software	NA.
Cell population abundance	NA.
Gating strategy	NA.

☐ Tick this box to confirm that a figure exemplifying the gating strategy is provided in the Supplementary Information.

Magnetic resonance imaging

Experimental design

Design type	NA.
Design specifications	NA.
Behavioral performance measures	NA .

Acquisition

Imaging type(s)	NA.
Field strength	NA.
Sequence & imaging parameters	NA.
Area of acquisition	NA.

Diffusion MRI ☐ Used ☐ Not used

Preprocessing

Preprocessing software	NA.
Normalization	NA.
Normalization template	NA.
Noise and artifact removal	NA.
Volume censoring	NA.

Statistical modeling & inference

Model type and settings	NA.
Effect(s) tested	NA.

Specify type of analysis: ☐ Whole brain ☐ ROI-based ☐ Both

Statistic type for inference<br>(See Eklund et al. 2016)	*NA.*
Correction	*NA.*

Models & analysis

n/a	Involved in the study
☒	☐ Functional and/or effective connectivity
☒	☐ Graph analysis
☒	☐ Multivariate modeling or predictive analysis

Functional and/or effective connectivity	*NA.*
Graph analysis	*NA.*
Multivariate modeling and predictive analysis	*NA.*
